# PAX3-FOXO1 Contacts BRD4 through Its Acetylated Intrinsically Disordered Region

**DOI:** 10.1021/acs.biochem.6c00040

**Published:** 2026-06-12

**Authors:** Olivia A. Fraser, Madeline N. Schleicher, Maya L. Pagano, Scott A. Showalter

**Affiliations:** † Department of Chemistry, The Pennsylvania State University, University Park, Pennsylvania 16802, United States; ‡ Center for Eukaryotic Gene Regulation, Department of Biochemistry and Molecular Biology, The Pennsylvania State University, University Park, Pennsylvania 16802, United States

## Abstract

Intrinsically disordered regions (IDRs) of transcription factors are frequent sites for post-translational modifications (PTMs), which mediate regulation through diverse mechanisms including protein–protein interactions. In the fusion oncoprotein PAX3-FOXO1, which drives alveolar rhabdomyosarcoma, a lysine-rich region of the FOXO1 IDR is subject to acetylation resulting in stabilization and enhanced transcriptional activity. Here, we leveraged ^13^C direct-detect nuclear magnetic resonance (NMR) spectroscopy to characterize acetylation in this system and identified a novel acetylation site corresponding to lysine 233 in endogenous FOXO1. In previous structural characterization of the endogenous FOXO1 DNA binding domain, local structure appears to prevent this site from becoming acetylated, suggesting that it becomes exposed in the context of the fusion protein. In addition, we demonstrate that the first bromodomain of the bromodomain and extraterminal domain-containing protein BRD4 binds to the acetylated region of interest and that this interaction is inhibited through the bromodomain and extraterminal domain inhibitor JQ1. These findings confer molecular mechanistic detail to previous observations that BRD4 and PAX3-FOXO1 colocalize at superenhancers in ARMS, adding to the growing body of literature exploring how BRD4 contacts cancer-relevant transcription factors in ways potentially relevant to the use of bromodomain and extraterminal domain inhibitors in cancer treatment.

## Graphical abstract

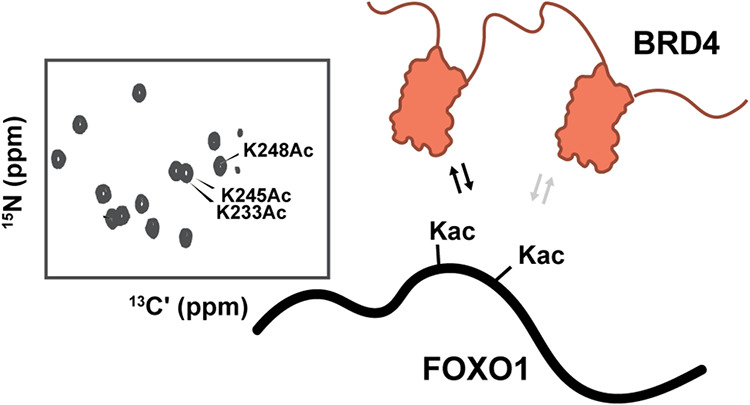

## 1 Introduction

The chemical information encoded in the primary structure of intrinsically disordered regions (IDRs) can be tuned through covalent modification, enabling dynamic control of protein function; understanding of mechanisms associated with regulation through post-translational modifications can be leveraged to understand or treat disease.^1,2^ For example, lysine acetylation in the C-terminal IDR of the transcription factor Forkhead Box O1 (FOXO1) plays a key role in regulation of FOXO1 target genes.^3–5^ This same region of FOXO1 is also translocated to PAX3-FOXO1 or PAX7-FOXO1 fusion oncoproteins found in a subset of patients (∼80%)^6^ with a rare and aggressive pediatric cancer, alveolar rhabdomyosarcoma (ARMS),^7–10^ and when present drives disease.^11,12^ Notably, while acetylation in the FOXO1-derived region of the fusion oncoprotein increases transcriptional activity at target genes, including oncogenes, in native FOXO1, acetylation of the identical region decreases DNA binding affinity.^13^ This reveals that acetylation in identical amino acid sequence contexts can regulate transcription by distinct yet undefined mechanisms.

In more than half of ARMS patients,^6^ the fusion oncoprotein PAX3-FOXO1 is formed by chromosomal translocation, resulting in the fusion of the DNA binding domains from the myogenic transcription factor Paired Box 3 (PAX3) to the C-terminal IDR of FOXO1, which replaces the native PAX3 IDR (Figure 1). While a complete mechanistic understanding of how replacement of the endogenous PAX3 IDR with the FOXO1-derived IDR drives disease progression remains undefined, it has previously been observed that PAX3-FOXO1 activates gene expression of myogenesis and oncogenesis factors through binding to distal enhancers and engaging transcriptional coactivators, such as the acetyllysine reader bromodomain-containing protein 4 (BRD4).^14–16^ In its best studied role, BRD4 promotes transcriptional elongation by recruiting the positive transcription elongation factor b (P-TEFb), which phosphorylates RNA Pol II and stimulates productive elongation.^17,18^ Additionally, an abundance of BRD4 has been observed at clusters of enhancers with strong activity, called superenhancers, in various cancers.^19–21^ The presence of BRD4 is associated both with acetyl marks on the histone tails, notably histone 3 lysine 27 acetylation, and nonhistone substrates such as transcription factors.^17–19, 22^

**Figure 1:**
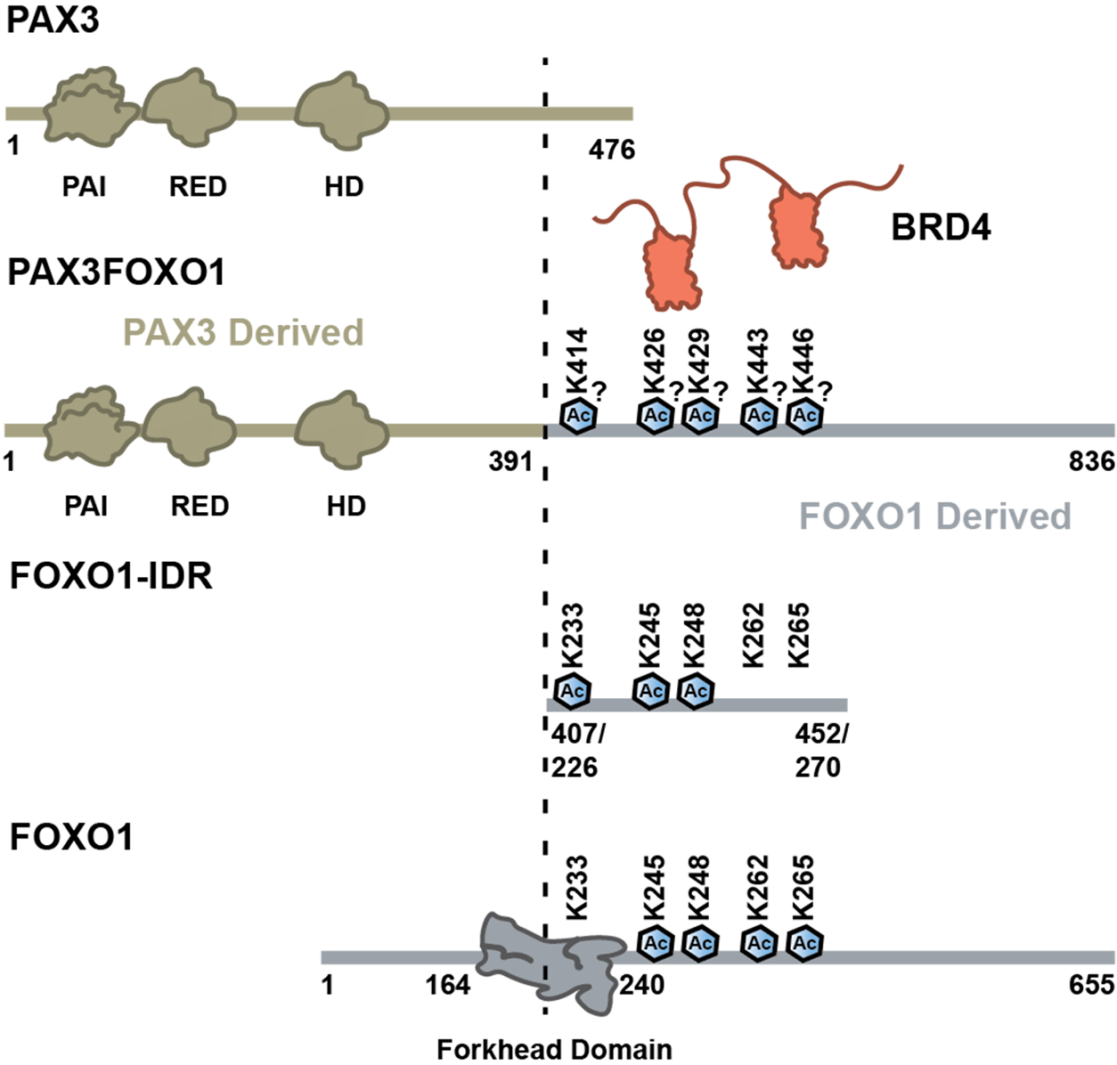
A schematic of the fusion oncoprotein PAX3FOXO1, which is comprised of the PAX3-derived DNA binding domains (PAIRED and homeodomain), and the FOXO1-derived IDR. Both contain key sites for post translational modification, notably acetylation C-terminal to the fusion junction. Acetylated lysine residues may be accessible for BRD4 binding, as investigated herein. The four literature-reported acetylation sites in native FOXO1 are marked with blue acetylation markers. In the context of the fusion protein, five FOXO1-derived lysine residues have the potential (annotated by question marks) to undergo acetylation by p300. The FOXO1-IDR segment investigated here is displayed between the PAX3FOXO1 fusion and native FOXO1 for reference. Note that for simplicity of the reader to locate sequence context from sources such as UniProt, all subsequent references to residue numbers will be relative to endogenous human FOXO1.

In ARMS-patient derived cell lines, PAX3-FOXO1 has been found to co-occupy superenhancers with BRD4, MED1, and p300.^16^ Notably, p300 is also the native acetyltransferase enzyme for endogenous FOXO1,^3^ motivating its use in the experiments described herein. Additionally, BRD4 has been implicated in maintaining stability of the oncoprotein in a manner that can be disrupted through treatment with a bromodomain and extraterminal domain inhibitor.^16^ BRD4 binding to acetylated lysine residues is mediated by its bromodomains and is most often studied in the context of its interaction with histones.^23,24^ As stated above, BRD4 bromodomains bind to acetylated lysine residues, where they facilitate chromatin organization and the formation of enhancer-promoter pairs to regulate transcription.^25,26^ However, transcription factors are also frequent nonhistone targets for lysine acetylation.^27,28^ Research on BRD4 interacting with the acetylated lysine residues of transcription factors lags equivalent research on their interactions with histones, with very few published examples of direct binding at the time of writing. One example is the RelA subunit of NF-κb, which is acetylated on lysine 310 by p300 before binding to BRD4. Subsequently, BRD4 recruits P-TEFb and RNAPII, just as it does after interacting with histones.^29^ The frequency of lysine acetylation on transcription factors, mediated by p300/CBP, GCN5, and other lysine acetyltransferase enzymes suggests that BRD4 could have many other binding targets, crucial to transcription.^22^ Gaining insight into these mechanisms is essential for a deeper understanding of transcription-regulating processes and would provide more developed explanations for the varied clinical success of BET inhibitors.

At present, the best evidence of interaction between BRD4 and PAX3-FOXO1 comes from rechromatin immunoprecipitation followed by sequencing (re-CHIP seq) assays, with further experimental evidence suggesting the interaction is mediated by one or more of the BRD4 bromodomains, although this has not been rigorously verified as a direct interaction.^16^ While the previously identified lysine-rich patch on PAX3-FOXO1 is the most likely region to mediate binding, this too has not been directly verified. We previously developed ^13^C direct-detect NMR spectroscopic methods to observe lysine acetylation, exploiting isotopically labeled ^13^C acetyl CoA as a substrate for lysine acetyl transferase enzymes, bypassing the need for uniform isotopic enrichment of the peptide substrate and enabling direct observation of PTMs in solution.^30,31^ Using this platform and ^13^C direct-detect methods that enable visualization of the peptide on an amino acid level.^32^ We characterized *in vitro* acetylation events in the FOXO1-derived IDR and examined its binding to the bromodomains of BRD4. These studies reveal direct binding between the acetylated C-terminal region and BRD4 through its first bromodomain. Implications for acetylation of the FOXO1-derived IDR in the PAX3-FOXO1 fusion protein will be discussed.

## 2 Materials and Methods

### 2.1 FOXO1 226–270 (FOXO1 IDR)

#### 2.1.1 Plasmid

The plasmid coding for the *Escherichia coli* codon-optimized *Homo sapiens* forkhead box protein O1 (FOXO1) in pET49b­(+) backbone was obtained from Genscript. The plasmid was altered to generate a protein encoding FOXO1 226–270 (see Uniprot Q12278), preceded by a fusion tag encoding GST, 6X-His, and a 3C protease cut site. Following protease cleavage of the expressed protein, this system leaves an N-terminal GPG peptide artifact derived from cloning. Acknowledging that this region encodes the N-terminal portion of the much larger full-length activation domain of FOXO1, this construct will be referred to as the FOXO1 IDR going forward. The plasmid was altered to enable production of a FOXO1 IDR variants containing the individual lysine to arginine substitutions K233R, K245R, K248R, K262R, and K265R using site-directed mutagenesis (New England Biolabs (NEB), 0552S).

#### 2.1.2 Protein Expression with Natural Isotope Abundance

Both the wild-type FOXO1 IDR and FOXO1 IDR variants were expressed and purified using an identical protocol. A 5 mL culture of BL21­(DE3) Fhu2A-*E. coli* (NEB, C2527I) transformed with the FOXO1 IDR plasmid was grown in Luria broth (LB) containing 50 μg/mL kanamycin at 37 °C for between 8 and 10 h.

500 μL of turbid 5 mL culture was used to inoculate 50 mL of LB supplemented with 50 μg/mL kanamycin. Growth proceeded for 10 h to saturation. Subsequently, 1 L LB supplemented with 50 μg/mL kanamycin was inoculated with the appropriate volume of the turbid 50 mL culture to achieve an initial OD_600_ of ∼0.1, typically 25–30 mL, and grown at 37 °C to an OD600 of ∼0.7. Expression was initiated with 0.5 mM isopropyl β-d-1-thiogalactopyranoside (IPTG). Following a 3 h expression at 37 °C, the cells were harvested by centrifugation at 3,400*g* at 4 °C and washed with 35 mL of phosphate buffered saline and either purified immediately or stored at −80 °C.

#### 2.1.3 Protein Expression with Uniform ^13^C, ^15^N Isotopic Enrichment

500 μL of turbid 5 mL culture (prepared identically to the above) was used to inoculate 50 mL of M9 minimal media supplemented with 1 g/L ^15^N-ammonium chloride (Cambridge Isotope Laboratories (CIL), DNLM-8739-PK), 2 g/L of ^13^C6-glucose (CIL, CLM-1396–1), 1× MEM vitamins (Corning, 25025CI), 1 mM MgSO_4_, 50 μg/mL kanamycin and 1× trace metals (Teknova, T1001). Growth proceeded for 10 h to saturation. Subsequently, 1 L of the M9 media supplemented as described was inoculated with the entire turbid 50 mL culture to a typical initial OD600 of 0.05–0.1 and grown at 37 °C to an OD600 of ∼0.7. Expression was initiated with 0.5 mM IPTG. Following a 3 h expression at 37 °C, the cells were harvested by centrifugation at 3,400*g* at 4 °C. and washed with 35 mL of phosphate buffered saline and either purified immediately or stored at −80 °C.

#### 2.1.4 Protein Purification

Cells containing recombinantly expressed FOXO1 IDR were lysed by sonication in 35 mL total volume of 50 mM Tris, 20 mM imidazole, 500 mM sodium chloride, 1 mM β-mercaptoethanol (BME), 1 mM phenylmethylsulfonyl fluoride, and 100 μL protease inhibitor cocktail set V, EDTA-free (Millipore Sigma, 539137–10VL) (50% duty cycle, 50% amplitude). Lysed cells were cleared by centrifugation at 14,000*g* for 30 min at 4 °C.

The decanted supernatant was passed through a 5 μm syringe filter before addition to a 15 mL bed volume nickel chelating resin (G-biosciences 786–407). The resin was washed with 45 mL of 50 mM Tris, 20 mM imidazole, 500 mM sodium chloride, 1 mM BME and eluted with 20 mL 50 mM Tris, 100 mM imidazole, 500 mM sodium chloride, 1 mM BME. 3C protease was used to cleave the GST and 6X-His tags concurrently with exchange using 1 kDa dialysis tubing (SpectraPor prewetted 132638) at 4 °C to 50 mM Tris, pH 9.5 (at 4 °C), 100 mM sodium chloride, 1 mM BME for 12 h. The clarified supernatant was brought to 65 °C in a water bath then incubated on ice until reaching a temperature of 10 °C or lower. The product was clarified by centrifugation at 14,000*g* for 25 min at 4 °C.

Dialysate was loaded onto a 15 mL bed volume SP sepharose (Cytiva, 17072910) gravity column previously equilibrated with 50 mM Tris pH 9.5 (at 4 °C), 100 mM NaCl, 1 mM BME. The protein was eluted using the loading buffer supplemented with a step gradient of 250–1000 mM sodium chloride (15 mL/fraction), where the 500 mM fraction contained the protein of interest as determined by SDS-PAGE (Tris-Tricine, 16%), visualized with both 2,2,2-trichloroethanol and Coomassie stain. The fraction containing the protein of interest was subsequently exchanged against 2L of 50 mM potassium phosphate, pH 7.2, 150 mM potassium chloride, using 1 kDa cutoff dialysis tubing, in preparation for storage. The dialysate was then concentrated using a 3KDa MWCO Amicon centrifugal filter. Protein was quantified by absorbance at 280 nm (ε_280_ = 11,000 M^–1^ cm^–1^). The expected yield from purification of protein recombinantly expressed in 1 L of media is 0.2 mmol (1 mg).

### 2.2 BRD4 BD1

#### 2.2.1 Plasmid

The plasmid containing *H. sapiens* BRD4 BD1 (40–170), Uniprot OP60885, was a gift from Dr. Song Tan (PSU) (Vector: Addgene #63935).^33^ In this plasmid, the open reading frame begins with a 6X-His tag and tobacco etch virus protease recognition site, such that cleavage of the resulting protein leaves an N-terminal SNA artifact. The plasmid was transformed into BL21­(DE3)­Fhu2A-*E. coli* (NEB, C25271I).

#### 2.2.2 Protein Expression

A 5 mL culture of BL21­(DE3)­Fhu2A-*E. coli* transformed with the BRD4 BD1 plasmid was grown in Luria broth (LB) containing 50 μg/mL kanamycin at 37 °C for between 8 and 10 h. 500 μL of turbid 5 mL culture was used to inoculate 50 mL of LB supplemented with 100 μg/mL ampicillin. Growth proceeded for 10 h to saturation. Subsequently, 1 L LB supplemented with 100 μg/mL ampicillin was inoculated with the appropriate volume of the turbid 50 mL culture to achieve an initial OD600 of ∼0.1, typically 25–30 mL, and grown at 37 °C to an OD600 of ∼0.7. Expression was initiated with 0.5 mM isopropyl β-d-1-thiogalactopyranoside (IPTG). Following a 16 h expression at 23 °C, the cells were harvested by centrifugation at 3,400*g* at 4 °C and washed with 35 mL of phosphate buffered saline and either purified immediately or stored at −80 °C.

#### 2.2.3 Protein Purification

Cells containing recombinantly expressed BRD4 BD1 were lysed by sonication in 35 mL total volume of 50 mM Tris, 20 mM imidazole, 500 mM sodium chloride, 1 mM β-mercaptoethanol (BME), 1 mM phenylmethylsulfonyl fluoride and 100 μL protease inhibitor cocktail set V, EDTA-free (Millipore Sigma, 539137–10VL) (50% duty cycle, 50% amplitude). Lysed cells were cleared by centrifugation at 14,000*g* for 30 min at 4 °C.

The supernatant was passed through a 5 μm syringe filter before addition to a 15 mL bed volume nickel chelating resin (G-biosciences 786–407). The resin was washed with 45 mL of 50 mM Tris, 20 mM imidazole, 500 mM sodium chloride, 1 mM BME and eluted with 20 mL of 50 mM Tris, 100 mM imidazole, 500 mM sodium chloride, 1 mM BME. Tobacco etch virus protease was used to cleave the N-terminal 6X-His tags concurrently with exchange using 3.5 kDa dialysis tubing (SpectraPor S632724) at 4 °C to 50 mM Tris, 20 mM imidazole, 500 mM sodium chloride, 1 mM BME (pH 7.5 at room temperature) for 12 h.

The dialysate was added to a 15 mL bed volume nickel chelating resin, where the protein is expected to elute in the flow-through. The resin was washed with 45 mL of 50 mM Tris, 20 mM imidazole, 500 mM sodium chloride, 1 mM BME, the remaining impurities and the 6X His tag were eluted with 20 mL of 50 mM Tris, 100 mM imidazole, 500 mM sodium chloride, 1 mM BME.

After analysis by SDS-PAGE, if any contaminants remained, the concentrated flow through from the nickel chelating resin step was passed over Sephacryl S-100 (Cytiva, 17061210) in 50 mM Tris pH 7.5, 150 mM NaCl, 1 mM DTT. Fractions containing BRD4 BD1 were determined by absorbance at 280 nm and SDS-PAGE. They were subsequently codialyzed alongside acetylated FOXO1 IDR to 50 mM potassium phosphate, pH 7.2, 150 mM potassium chloride in a 2L solution. The expected yield from purification of protein recombinantly expressed in 1 L of media was 0.19 mmol (3 mg).

### 2.3 BRD4 BD2

#### 2.3.1 Plasmid

The plasmid containing *H. sapiens* BRD4 BD2 (349–472) was a gift from Dr. Song Tan (PSU) (Vector: Addgene #63935).^33^ In this plasmid, the open reading frame begins with a 6X-His tag and tobacco etch virus protease recognition site, such that cleavage of the resulting protein leaves an N-terminal SNA artifact. The plasmid was transformed into BL21­(DE3)­Fhu2A-*E. coli* (NEB, C25271I).

#### 2.3.2 Expression

A 5 mL culture of BL21­(DE3)­Fhu2A-*E. coli* transformed with the BRD4 BD2 plasmid was grown in Luria broth (LB) containing 50 μg/mL kanamycin at 37 °C for between 8 and 10 h. 500 μL of turbid 5 mL culture was used to inoculate 50 mL of LB supplemented with 100 μg/mL ampicillin. Growth proceeded for 10 h to saturation. Subsequently, 1 L LB supplemented with 100 μg/mL ampicillin was inoculated with the appropriate volume of the turbid 50 mL culture to achieve an initial OD600 of ∼0.1, typically 25–30 mL, and grown at 37 °C to an OD600 of ∼0.7. Expression was initiated with 0.5 mM isopropyl β-d-1-thiogalactopyranoside (IPTG). Following a 3 h expression at 37 °C, the cells were harvested by centrifugation at 3,400*g* at 4 °C and washed with 35 mL of phosphate-buffered saline and either purified immediately or stored at −80 °C.

#### 2.3.3 Purification

Cells containing recombinantly expressed BRD4 BD2 were lysed by sonication in 35 mL total volume of 50 mM Tris, 20 mM imidazole, 500 mM sodium chloride, 1 mM β-mercaptoethanol (BME), 1 mM phenylmethylsulfonyl fluoride and 100 μL protease inhibitor cocktail set V, EDTA-free (Millipore Sigma, 539137–10VL) (50% duty cycle, 50% amplitude). Lysed cells were cleared by centrifugation at 14,000*g* for 30 min at 4 °C.

The supernatant was passed through a 5 μm syringe filter before addition to a 15 mL bed volume nickel chelating resin (G-biosciences 786–407). The resin was washed with 45 mL of 50 mM Tris, 20 mM imidazole, 500 mM sodium chloride, 1 mM BME and eluted with 20 mL of 50 mM Tris, 200 mM imidazole, 500 mM sodium chloride, 1 mM BME. Tobacco etch virus protease was used to cleave the N-terminal 6X-His tags concurrently with exchange using 3.5 kDa dialysis tubing (SpectraPor S632724) at 4 °C to 50 mM Tris, 20 mM imidazole, 500 mM sodium chloride, 1 mM BME (pH 7.5 at room temperature) for 12 h.

The dialysate was added to a 15 mL bed volume nickel chelating resin, where the protein is expected to elute in the flow-through. The resin was washed with 45 mL of 50 mM Tris, 20 mM imidazole, 500 mM sodium chloride, 1 mM BME, the remaining impurities and the 6X His tag were eluted with 20 mL of 50 mM Tris, 200 mM imidazole, 500 mM sodium chloride, 1 mM BME. After analysis by SDS-PAGE, BRD4 BD2 was quantified by measuring absorbance at 280 nm. Expected yield was 0.1 μmol (1.3 mg).

### 2.4 P300 KAT

#### 2.4.1 Plasmid

The lysine acetyltransferase p300 (Uniprot Q09472) was prepared from a plasmid previously generated by Dr. S. Dewing (Addgene #233588),^34^ which encodes p300 lysine acetyltransferase (KAT) domain optimized for downstream *in vitro* acetylation reactions through deletion of the activation loop. The construct was transferred to the 1 M vector using ligation-independent cloning (Addgene #29656), in which it expressed with an N-terminal fusion encoding maltose-binding protein, a 6X-His tag, and a tobacco etch virus protease cleavage site, conferring an N-terminal SNA peptide artifact derived from cloning. The resulting plasmid was transformed into Rosetta 2­(DE3)-*E. coli* competent cells (Millipore Sigma 71400-M).

#### 2.4.2 Expression

A 100 mL culture of Rosetta 2­(De3)-*E. coli* transformed with the p300 KAT plasmid was grown in Luria broth (LB) containing 50 μg/mL kanamycin and 25 μg/mL of chloramphenicol at 37 °C for between 8 and 10 h. 25 mL of turbid 100 mL culture was used to inoculate 1 L of Terrific Broth (TB) supplemented with 50 μg/mL kanamycin, 25 μg/mL chloramphenicol, and 0.4% w/v glycerol. Growth proceeded for 10 h to a typical optical density at 600 nm (OD_600_) of 0.9. Expression was initiated with 0.5 mM isopropyl β-d-1-thiogalactopyranoside (IPTG). Following a 18 h expression at 18 °C, the cells were harvested by centrifugation at 3,400*g* at 4 °C and washed with 35 mL of phosphate-buffered saline and stored at −80 °C.

#### 2.4.3 Purification

All purification steps were done at either 4 °C or on ice. Cells containing recombinantly expressed p300 KAT were lysed by sonication in 35 mL total volume of 50 mM HEPES pH = 7.0, 30 mM imidazole, 500 mM sodium chloride, 5 mM β-mercaptoethanol (BME), 1 mM phenylmethylsulfonyl fluoride (PMSF), 200 μL protease inhibitor cocktail set V, EDTA-free (PIC)­(Millipore Sigma, 539137–10VL), and 10% w/v glycerol (50% duty cycle, 50% amplitude). Lysed cells were cleared by centrifugation at 18,000*g* for 40 min at 4 °C.

The supernatant was passed through a 5 μm syringe filter before addition to a 15 mL bed volume nickel chelating resin (G-biosciences 786–407). The resin was washed with 45 mL of 50 mM HEPES pH = 7.0, 30 mM imidazole, 1 M sodium chloride, 5 mM BME, 1 mM PMSF, and 100 μL PIC, and 10% w/v glycerol. The resin was washed again with 45 mL of 50 mM HEPES pH = 7.0, 30 mM imidazole, 150 mM sodium chloride, 5 mM BME, 1 mM PMSF, and 100 μL PIC, and 10% w/v glycerol. 6X-His tagged p300 KAT eluted with 20 mL of 50 mM HEPES pH= 7.0, 300 mM imidazole, 150 mM sodium chloride, 5 mM BME, 1 mM PMSF, 100 μL pIC, and 10% w/v glycerol. Tobacco etch virus protease was used to cleave the N-terminal 6X-His tags concurrently with exchange using 3.5 kDa dialysis tubing (SpectraPor S632724) at 4 °C to 50 mM HEPES pH = 7.0, 30 mM imidazole, 150 mM sodium chloride, 5 mM BME, 1 mM PMSF, 10% w/v glycerol for 12 h.

The dialysate was added to a 15 mL bed volume nickel chelating resin, where the protein is expected to elute in the flow through. The resin was washed with 45 mL of 50 mM HEPES pH = 7.0, 30 mM imidazole, 150 mM sodium chloride, 5 mM BME, 1 mM PMSF, and 10% w/v glycerol. Remaining impurities and the 6X His tag eluted with 20 mL 50 mM HEPES pH = 7.0, 300 mM imidazole, 150 mM sodium chloride, 5 mM BME, 1 mM PMSF, 10% w/v glycerol. After analysis by SDS-PAGE, p300 KAT was quantified by measuring absorbance at 280 nm. Expected yield was 0.18 μmol (8.1 mg). Purified p300 KAT was stored in 200 μM aliquots at −80 °C.

### 2.5 ^13^C Acetyl CoA Synthesis

CoA lithium salt (1 equiv, CoALA Biosciences, AC02) and 1,1′,2,2′-^13^C acetic anhydride (1.8 equiv, Cambridge Isotope Laboratories, CLM-1161–1) were combined in 200 μL 0.5 M sodium bicarbonate in a capped microcentrifuge tube and incubated on ice for 45 min.^30,35^ The product was stored at −80 °C without further purification.

### 2.6 Enzymatic Transfer of ^13^C′,^13^C^ali^ Acetyl for Resonance Assignments

#### 2.6.1 Resonance Assignment Sample Acetylation

Acetyl transfer from 600 μM ^13^C′,^13^C^ali^ acetyl CoA to 50 μM ^13^C, ^15^N enriched FOXO1 IDR was carried out in a 12 mL reaction in 50 mM Tris/50 mM BisTris, 20 mM sodium acetate, 1 mM DTT, pH 7.5, initiated by the addition of 1 μM p300. The reaction proceeded at 25 °C for 24 h. Following this, the reaction was incubated at 80 °C for 5 min to heat inactivate the enzyme and pelleted by centrifugation at 16,000*g* for 10 min. The supernatant was decanted and buffer exchanged to 50 mM potassium phosphate, 150 mM potassium chloride, pH 7.2 in 1 kDa dialysis tubing (SpectraPor, 132638), then concentrated to 500 μL (final concentration of 1.2 mM) using a 3 kDa MWCO Amicon centrifugal filter. D_2_O (10%) 1 mM DTT, and sodium azide were added to the sample. The resultant sample was transferred to a 5 mm NMR tube (Wilmad Lab Glass 528-PP-7).

#### 2.6.2 Peptide Acetylation

Peptides from *H. sapiens* FOXO1­(Uniprot Q12778) and FOXO3 (Uniprot O43524) 240–255 were ordered as lyophilized powders from Tufts core facility. These were dissolved at 3 mM in 50 mM Tris, 50 mM Bis-tris, 20 mM Sodium acetate, 1 mM DTT. Acetylation reactions for each peptide were carried out in 500 μL with 1.25 mM of peptide, 15 mM Acetyl CoA, 25 μM p300. This yielded a majority +2 acetylation state of K245ac and K248ac for FOXO1, or K242ac K245ac for Foxo3.

#### 2.6.3 Binding Sample Acetylation

Acetyl transfer was carried out using identical conditions to those used to generate the resonance assignment sample, with the exception that the total reaction volume was 4.5 mL. Following the heat inactivation step, the supernatant was decanted and buffer exchanged to 50 mM potassium phosphate, 150 mM potassium chloride, pH 7.2 in 1 kDa dialysis tubing (SpectraPor, 132638) and codialyzed in the same reservoir as the BRD4 BD1 and BD2 preparations to ensure an exact buffer match, then concentrated to a final concentration of 350 μM using a 3 kDa MWCO Amicon centrifugal filter. D_2_O (10%) was added to the sample. The resultant sample was transferred to a 5 mm NMR tube (Wilmad Lab Glass 528-PP-7).

### 2.7 Bromodomain Binding

NMR samples contained 350 μM FOXO1 with either BRD4 BD1 or BD2 at equimolar concentration supplemented with 10% D_2_O and were loaded into 5 mm Shigemi tubes matched for 500 MHz field strength (Wilmad Lab Glass BMS-005B). JQ1 was dissolved in DMSO to a 100 mM stock due to insolubility in aqueous buffers, a concentration chosen such that spike-in of JQ1 would not exceed >1% DMSO in the final solution. Three further sample conditions were prepared: (i) 350 μM FOXO1 in 0.5% DMSO; (ii) 350 μM FOXO1 with 350 μM BRD4 BD1 and 700 μM JQ1 (final DMSO 0.5%); and (iii) 350 μM FOXO1 with 700 μM JQ1 (final DMSO 0.5%). Samples were loaded into 5 mm Shigemi tubes matched for 500 MHz field strength (Wilmad Lab Glass BMS-005B).

NMR samples containing 100 μM BRD4 BD1 or BRD4 BD2 in 50 mM potassium phosphate and 150 mM potassium chloride, pH = 7.2, were supplemented with 10% D_2_O. [^1^H,^15^N]-HSQC spectra were collected for each unbound bromodomain before adding 5 mol equiv of either biacetylated FOXO1 or FOXO3 peptide residues 240–255.

### 2.8 Nuclear Magnetic Resonance

NMR spectra were collected at 25 °C on either a Bruker Avance NEO 600 MHz spectrometer (the (HACA)­N­(CA)­NCO experiment) or Bruker AVIII 500 MHz spectrometer (all other experiments), both equipped with 5 mm TCI triple-resonance cryoprobes.

#### 2.8.1 Resonance Assignment

Amino acid connectivity was established using 3D (HACA)­N­(CA)­CON and (HACA)­N­(CA)­NCO experiments and the amino acid identity of the resonance was established by the 3D (H)­CCCON experiment as per standard protocols.^32^ On-instrument data processing was performed using Bruker Topspin (for (HACA)­N­(CA)­NCO, version 4.4.0; all else, version 3.2.6). All NMR data, apart from the (HACA)­N­(CA)­NCO experiments, were acquired using 8 scans per increment, 1024 complex points in the direct dimension and 256 increments in the indirect dimension. With these parameters, the total acquisition times were 51 h for 3D (HACA)­N­(CA)­CON, 49 h for 3D (H)­CCCON, and 100 h for 3D (HACA)­N­(CA)­NCO. The spectral widths were 19 ppm (^13^C′) and 47 ppm (^15^N), centered on 173.9 and 123.4 ppm, respectively. Spectra were visualized and backbone resonance assignment was conducted using NMRFAM-Sparky.^36^ Samples were referenced in processing to natural abundance sodium trimethylsilylpropanesulfonate (DSS), the ^13^C-methyl chemical shift of which was determined to be −2.6 ppm in a buffer only control ^13^C 1D spectrum.

To calculate chemical shift perturbations (CSP) upon acetylation, whitespace delimited text files of chemical shifts were extracted from NMRFAM-Sparky (saved by default with the extension “.shifts”) after backbone resonance assignment of the [^13^C′, ^15^N]-CON-IPAP. The magnitude of the chemical shift perturbation (CSP) between each acetylated resonance and its corresponding unacetylated resonance were calculated as

1 CSP=(ΔC13)2+(0.2·ΔN15)2

where Δ^13^C is the change in the carbonyl carbon chemical shift and Δ^15^N is the change in the amide nitrogen chemical shift. If multiple acetylated resonances could be assigned to a single residue due to sample homogeneity, the CSP of each acetylated resonance was referenced to the unacetylated chemical shifts for the same residue.

#### 2.8.2 Binding

Acetylated lysines were selectively visualized using the ^1^H-start [^13^C′,^13^C^ali^]-CaliCO-K_ac_-IPAP.^31^ NMR data were acquired using 8 scans per increment, 1024 complex points in the direct dimension and 128 increments in the indirect dimension. The spectral widths were 20 ppm (^13^C′) and 50 ppm (^13^C^ali^), centered on 173.9 and 25 ppm respectively, yielding an acquisition time of 1 h per experiment. On instrument data processing was conducted using Bruker Topspin version 3.2.6. Perturbations to the acetylated lysine upon binding to BRD4 BD1 or BD2, and upon supplementation with JQ1, were visualized using NMRFAM-Sparky.^36^

Bromodomain backbone perturbations from acetylated peptide binding were visualized using [^1^H,^15^N]-HSQC experiments. NMR data were acquired using 16 scans per increment, 2048 complex points in the direct dimension and 256 increments in the indirect dimension, yielding an acquisition time of 1.5 h per experiment. The spectral widths were 16 ppm (^1^H) and 28 ppm (^15^N), centered on 4.7 and 120 ppm, respectively. On instrument data processing was conducted using Bruker Topspin version 3.2.6. Perturbations to the bromodomain backbone resonances upon acetylated peptide binding were visualized using NMRFAM-Sparky.^36^

### 2.9 Mass Spectrometry

Matrix-assisted laser desorption/ionization-time-of-flight (MALDI-TOF) was used to collect mass spectra on unmodified FOXO1 IDR and acetylated FOXO1 IDR using an Ultraflextreme instrument (Bruker). Samples were brought to 50 μM and 50 μL aliquots were desalted with C-18 Spin Columns (G-Biosiences 786–930) followed by evaporation of solvent with centrifugation at 1400 rpm at 30 °C under vacuum for 3 h. FOXO1 IDR samples were resuspended in 5 μL of water and mixed 1:1 with matrix containing 20 mg/mL Super-DHB (Sigma-Aldritch 50862), 50% acetonitrile, 1% trifluoracetic acid, and 1% phosphoric acid.

## 3 Results and Discussion

### 3.1 Rationale and Approach

To characterize the putative FOXO1/BRD4 interaction, we focused on a segment of the C-terminal FOXO1 IDR located proximally to the forkhead domain in the native protein, known to undergo multiple lysine acetylation events that may serve as a mediator for the interaction, and which are retained in the fusion oncoprotein PAX3-FOXO1.^4^ In native FOXO1, acetylation in this region is sufficient to decrease affinity for cognate DNA, yet the structural features of the region remain unexplored, likely due to its intrinsic disorder.^13^ A Metapredict trace used to predict the level of disorder of the FOXO1 IDR construct in the context of PAX3-FOXO1 fusion protein matches its intrinsic disorder in the context of native FOXO1 (Figure S1).^37^ ^13^C direct-detect NMR spectroscopy was chosen to investigate this region on an amino-acid level due to its unique advantages in studying IDRs,^38,39^ namely that (i) it is a solution-based technique, (ii) it provides enhanced resolution over ^1^H-detected NMR spectroscopy in the case of IDRs, due to improved chemical shift dispersion, and (iii) its detection modality relies on the presence of a covalent carbonyl to nitrogen bond, which is present in the backbone of all amino acids, including proline.

### 3.2 Identification of In Vitro FOXO1 IDR Acetylation Events by p300

To enable our study, ^13^C/^15^N backbone resonance assignments were conducted using ^13^C direct-detect NMR spectroscopy for the unmodified and acetylated FOXO1 IDR. Assigned spectra are provided for unmodified FOXO1 IDR (Figure S2) and acetylated FOXO1 IDR (Figure S3). The full chemical shift set has been deposited in the BioMagResBank (unmodified Access Number 53507; acetylated Access Number 53508). We accessed a highly reproducible acetylation state where the predominant species were acetylated either two or three times, as confirmed by MALDI-TOF mass spectrometry (Figure S4). In the backbone optimized [^13^C′, ^15^N]-CON spectrum of acetylated FOXO1 IDR, the most significant chemical shift perturbations, relative to the unmodified FOXO1 IDR spectrum, are proximal to lysines 233, 245, and 248 (Figure 2A). Note that due to the mixture of double- and triple-acetylated species, some residues have more than one corresponding resonance in the acetylated spectrum, such as lysine 265. For most resonances which exhibit two assigned resonances, one is associated with a larger chemical shift perturbation, while the other is smaller and may correspond to the absence of local acetylation. The residue level CSPs are displayed in Figure 2B, with peak doubling indicated through split bars. While the regions displaying the largest CSPs are likely to correspond to those lysine residues that are acetylated, changes to the conformational ensemble can also be captured by this method, necessitating further chemical shift analysis to definitively identify the sites of acetylation.

**Figure 2:**
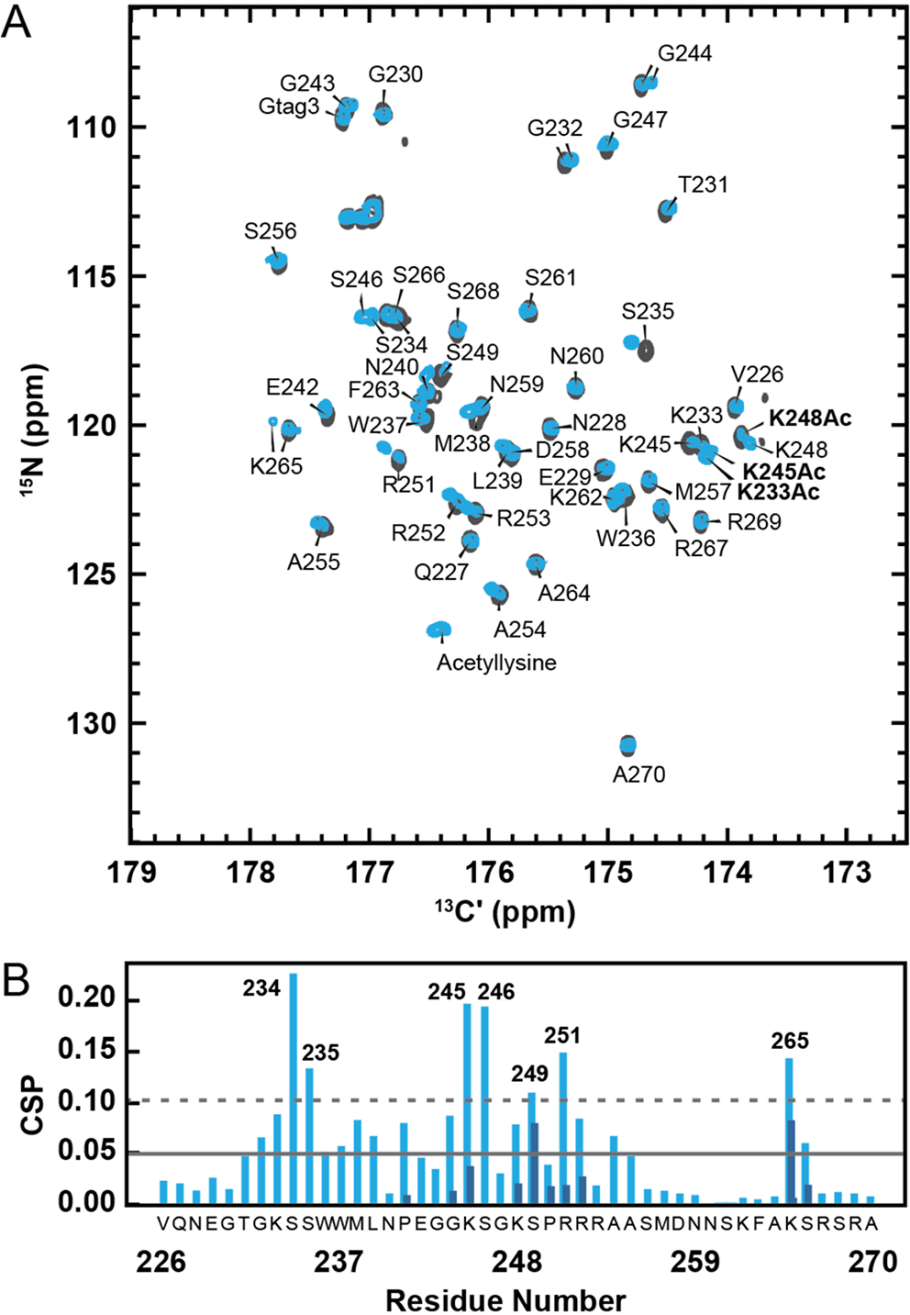
NMR observation of *in vitro* acetylation of the FOXO1 IDR by p300. (A) Overlayed [^13^C′, ^15^N]-CON spectra of unacetylated (gray) and acetylated (pale blue) FOXO1 IDR. Residue assignments are annotated to reflect the residue contributing the nitrogen atom to the C′N bond. Note that the spectrum has been zoomed in to the most populated region for clarity, and the full spectrum can be seen in Supporting Figure 1. (B) Chemical shift perturbations upon acetylation were quantified using eq 1, where the solid gray horizontal line represents the average perturbation and the dotted gray horizontal line represents the value of one standard deviation above the average. The residue number assigned to resonances which exhibited a chemical shift perturbation greater than one standard deviation above the average are labeled for clarity. For residues with more than one assigned corresponding resonance in the acetylated spectrum due to sample heterogeneity, the smaller CSP is displayed in dark blue.

Previous studies have reported chemical shift perturbations in the lysine δ-carbon resonance (from ∼29 ppm to ∼30.5 ppm), and smaller but still systematic perturbations in the ε-carbon chemical shift, that are diagnostic of lysine acetylation.^40,41^ Therefore, 3D (H)­CCCON-IPAP spectroscopy was used to completely determine the aliphatic side-chain carbon chemical shifts in FOXO1 IDR; here, we will emphasize analysis of the lysine resonances to establish which residues undergo the greatest level of acetylation by p300. The chemical shift patterns displayed in Figure 3 identify lysine 233, 245, and 248 as the primary sites of acetylation. K233 and its nearest sequential neighbors only produce one resonance in the [^13^C′, ^15^N]-CON, with chemical shifts that are distinct from the unmodified state, while the associated K233 ^13^Cδ in the (H)­CCCON shifts downfield to 30.5 ppm (Figure 3A). Therefore, we conclude that lysine 233 is acetylated stoichiometrically, or at least that the population of unmodified K233 is not sufficient for its resonance to appear above the baseline noise.

**Figure 3:**
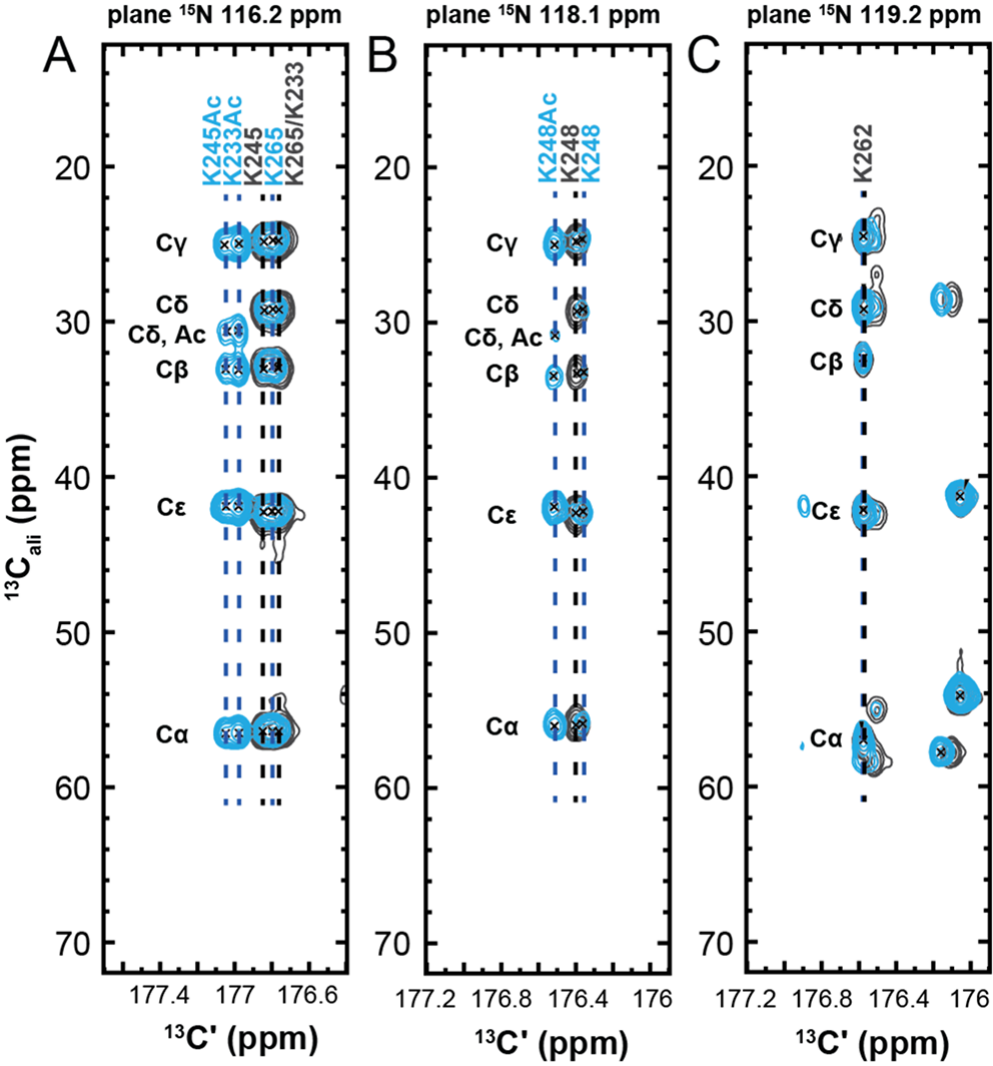
An overlay of planes from a three-dimensional (H)­CCCON-IPAP spectrum acquired on FOXO1 IDR, either unmodified (dark gray) or subjected to acetylation conditions (blue). (A) ^13^C_ali_, ^13^C′ plane centered on K233, K245, and K265. (B) ^13^C_ali_, ^13^C′ plane centered on K248. (C) ^13^C_ali_, ^13^C′ plane centered on K262. The ^15^N chemical shifts corresponding to each plane are annotated above the panel. A shift in the lysine δ-carbon resonance from ∼29 ppm to ∼30.5 ppm indicates acetylation and is observed for K233, K245, and K248.

The NMR data support partial acetylation of K245 and K248 by p300 *in vitro*, while K262 and K265 are not appreciably acetylated. Coupled with the stoichiometric acetylation of K233, this interpretation is consistent with the observation of a majority 2 or 3 acetyl-state by mass spectrometry with no residual unacetylated peptide remaining (Figure S2). The relative peak intensities of K245 and K245ac suggest that this residue is 52% acetylated, while the intensities for K248 and K248ac are consistent with 63% acetylation at this site. Downfield shifts of the ^13^Cδ resonances to ∼30.5 ppm in the 3D (H)­CCCON spectrum informs the acetylation state-assignment associated with each resonance in the [^13^C′, ^15^N]-CON. In both the K245 and K248 cases, the nearest sequential neighbors also split into two distinct [^13^C′, ^15^N]-CON resonances, with unambiguous sequential assignment achievable. It is statistically likely that our samples contain a mixture of FOXO1 molecules with zero, one, or two acetylated lysine residues in the K245/K248 region. However, there are no occurrences of three [^13^C′, ^15^N]-CON resonances being assigned to a single amino acid. This suggests that the K245/K248 double-acetylated state is not sufficiently populated to yield a resonance above the noise floor, which could be attributed to the low inherent sensitivity of [^13^C′, ^15^N]-CON spectroscopy, that the population of the single-acetyl intermediate is depleted due to sequential processivity of p300 to favor a mixture of the +0 and +2 acetyl state in this motif, or that the distance separating K245 and K248 is sufficiently large for their chemical shifts to be mutually insensitive to the acetylation status of the other.

In contrast, the [^13^C,^15^N]-CON resonances of K262 and K265 remain intense and no secondary set of resonances for these residues and their immediate sequential neighbors were identified. The (H)­CCCON spectrum reveals ^13^Cδ chemical shifts consistent with an unmodified side chain (Figure 3A,C). Note that, although the ^13^C,^15^N-CON resonances of K265 and unmodified K245 are partially overlapped, their resolution in the ^13^C′ dimension is sufficient to unambiguously interpret the acetylation status of both side chains in the 3D (H)­CCCON spectrum (Figure 3A); if there were a downfield shift to either residue’s ^13^Cδ resonance, it would be observable and attributable to the originating residue.

We next sought to assess the uniqueness of the chemical environments accessed by each acetyllysine side chain, as represented by the variation in the NMR chemical shifts of nuclei composing the carbon–nitrogen bond in the acetamide functional group. The spin state selective [^13^C′,^15^N]-CON-K_ac_-IPAP permits selective visualization of the acetyllysine resonance in the context of uniformly labeled peptide substrate.^30^ Thus, it is possible to unambiguously assign the resonance corresponding to the acetyllysine moiety. In addition, this experiment provides enhanced spectral resolution in comparison to the backbone optimized [^13^C′,^15^N]-CON (Figure S5). Notably, the high resolution achieved reveals at least two acetyllysine resonances, possibly three, that are only partially degenerate in their chemical shifts (Figure S5). This suggests that, even as far from the protein backbone as the Nε-acetylation, the chemical environments of each acetyllysine in FOXO1 IDR are subtly distinct, which may have implications for the selectivity of downstream protein–protein interactions. Further, this observation contrasts with our previous observations in histone H3 tail,^30^ where the observed acetyllysine resonances were fully degenerate in chemical shift.

As there are five lysine residues in the construct used, each of which is a possible p300 target, we undertook additional steps to ensure full and unambiguous assignment of each residue. The unique sequence context of K262 (preceded by a serine) and K265 (preceded by an alanine) aided in analysis, whereas K233, K245, and K248 are all preceded by glycine. The resonance assigned to the backbone nitrogen of K265 has a C′-1 chemical shift of 177.7 ppm, typical for an alanine (average: 177.747 ppm; all average chemical shifts reported here were accessed from biological magnetic resonance bank (BMRB) on June 13, 2025), and differentiable from the average C′ chemical shift for a glycine (173.832 ppm), which further strengthened our confidence in these assignments.^42^ Analysis of the chemical shift sets for the residue preceding each lysine definitively assigns K262 and K265, whereas the other three lysines are all preceded by a glycine residue (Figure S6). Unambiguous connectivity for each of lysine K233, K245, and K248 is achieved through analysis of adjacent nitrogen chemical shifts in the HACANCACON and HACANCANCO spectra.

We sought to establish additional rigor in our spectral assignments for K233 because prior work using limited proteolysis and mass spectrometry to identify p300 acetylation sites within the FOXO1 forkhead domain, which included the region examined here, did not observe modification at this site.^13^ We introduced a K233R substitution to unambiguously confirm the identity of this resonance. In the backbone optimized [^13^C′, ^15^N]-CON of FOXO1 IDR K233R, in addition to the loss of the assigned K233 resonance, a new resonance is visible (with chemical shifts 176.5 ppm, 116.6 ppm), consistent with the BMRB-average N of serine 234 (average: 116.304 ppm) and C′ of arginine 233 (average: 176.429 ppm). We further analyzed the side chain ^13^C chemical shifts corresponding to the new resonance located on this ^15^N plane, noting characteristic arginine chemical shifts (Figure 4). In constructs containing the forkhead domain, lysine 233 is in wing 1 of the forkhead domain of FOXO1.^13^ This evidence supports our assignment of lysine 233 as an *in vitro* target of p300 in the FOXO1-derived residues contributing to the PAX3-FOXO1 fusion protein, but not in the IDR as presented in native FOXO1, where this residue is found within the structured region of the forkhead domain. We thus hypothesize that K233 is differentially accessible to p300 in the context of the fusion protein compared to in the native FOXO1 context, where it is proximal to the structured forkhead domain, suggesting that the fusion may expose additional regulatory sites.

**Figure 4:**
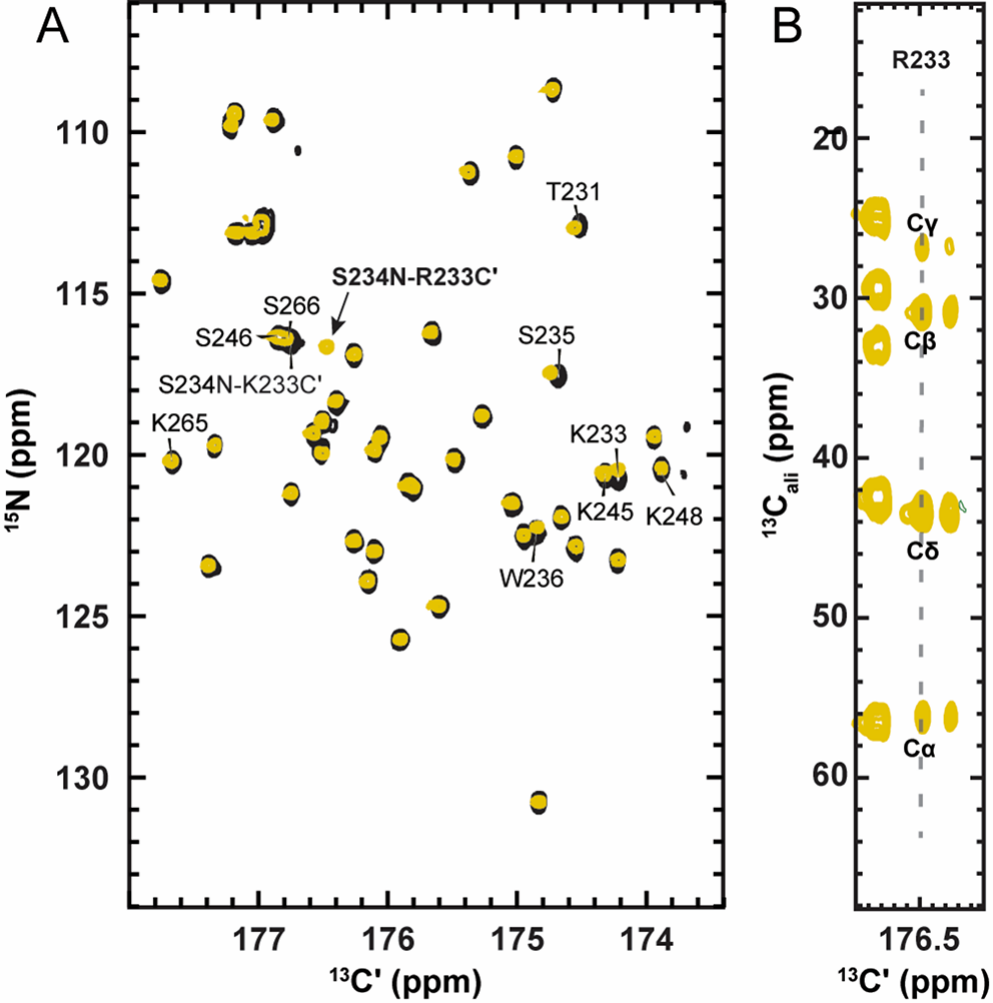
NMR spectra of the FOXO1 IDR K223R mutant confirm the accuracy of the K233 resonance assignment. (A) Overlay of the wild-type (black) and K233R (yellow) [^13^C′, ^15^N]-CON with key resonances annotated demonstrates clear perturbation of residues proximal in sequence to 233 and the disappearance of the S234–K233 resonance (emphasis through bold font and arrow). (B) (H)­CCCON spectral plane at 116.4 ppm ^15^N chemical shift ^13^C_ali_, ^13^C′ plane centered on, the S234 backbone resonance demonstrates the preceding residue ^13^C chemical shifts are consistent with an arginine.

To validate the claim that the region including K233 is unstructured when removed from the context of the forkhead domain, we calculated its secondary structure propensity in both the unmodified and acetylated state, using the deviation of experimentally derived Cα and Cβ chemical shifts from a data set of random coil chemical shifts^43^ (Figure S7). We detected no persistent secondary structure in the region surrounding the K233 acetylation site, supporting the conclusion that this residue is in an unstructured environment in the fusion oncoprotein. Furthermore, our analysis reveals no strong changes to secondary structure elements in response to acetylation, ruling this mechanism out as a driver of enhanced transcriptional activity in the context of FOXO1 or PAX3-FOXO1.

The assignments reported here may also serve as a reference for future studies by facilitating the assignment of larger FOXO1 constructs by transfer-based approaches. Such future constructs may include the folded forkhead domain of native FOXO1 with extended disordered regions and/or constructs spanning the fusion junction. Additionally, as this region includes a portion of endogenous FOXO1 known to be important for DNA binding but absent from the cocrystal structure of the FOXO1 forkhead domain bound to DNA,^13^ elucidation of the mechanism by which acetylation impacts FOXO1 binding to DNA will also be empowered by generation of assignments in this region.

### 3.3 BRD4 BD1 Binds Acetylated FOXO1 IDR

The bromodomain and extraterminal domain containing protein BRD4 colocalizes with PAX3-FOXO1 at superenhancers in ARMS patient-derived cell lines;^16^ thus, we chose to investigate the ability of the acetylated FOXO1 IDR to bind the first and second bromodomains of BRD4 (BD1 and BD2, respectively). Evidence that this interaction occurs through the bromodomains of BRD4 stems from the disruption of PAX3-FOXO1 stability upon treatment with a small molecule that inhibits bromodomain binding (JQ1). Native FOXO1 colocalizes with BRD4 bromodomains, and BRD4 affects FOXO1 transactivation abilities. For example, p21 expression is increased following BRD4 treatment of JQ1, suggesting the presence of BRD4-FOXO1 interaction that, when suppressed, is a potential therapeutic target for prostate cancer.^44,45^ Conversely, p63 expression is upregulated by the colocalization of FOXO1 and BRD4 in basal mammary epithelial cells, and downregulated when BRD4 is suppressed.^46^ Surprisingly few papers have employed recombinant proteins and biochemical methods to investigate the presumptive interaction between FOXO1 and BRD4, although one paper reporting on FOXO3 function does include [^1^H,^15^N]-NMR spectra confirming chemical shift perturbations in BRD4 bromodomains upon interaction with a FOXO1-derived peptide including K245ac and K248ac.^47^ Overall, the molecular details for how BRD4 engages PAX3-FOXO1 or FOXO1 remain unclear, despite the potential therapeutic avenues which could stem from interruption of the interaction.

Based on our prior observation that acetylated lysine residues 14 and 18 on the histone H3 tail resolved upon binding to the GCN5 bromodomain, we employed ^1^H-start [^13^C′,^13^C^ali^]-CaliCO-K_ac_-IPAP to probe binding of BRD4 BD1 and BD2 and acetylated FOXO1 IDR.^30^ Notably, observed perturbations to the acetyllysine resonance upon binding to BD1 resemble previous observations of histone H3 binding to the GCN5 bromodomain, which provides further evidence of a direct interaction (Figure 5). To probe if the interaction is mediated selectively through the acetyllysine binding pocket of the bromodomain, we also used the selective inhibitor JQ1 to compete for the binding pocket and observed return of the chemical shift back to that of unbound FOXO1 IDR. Thus, we note that the interaction between the acetylated FOXO1 region must be mediated through the BRD4 bromodomain.

**Figure 5:**
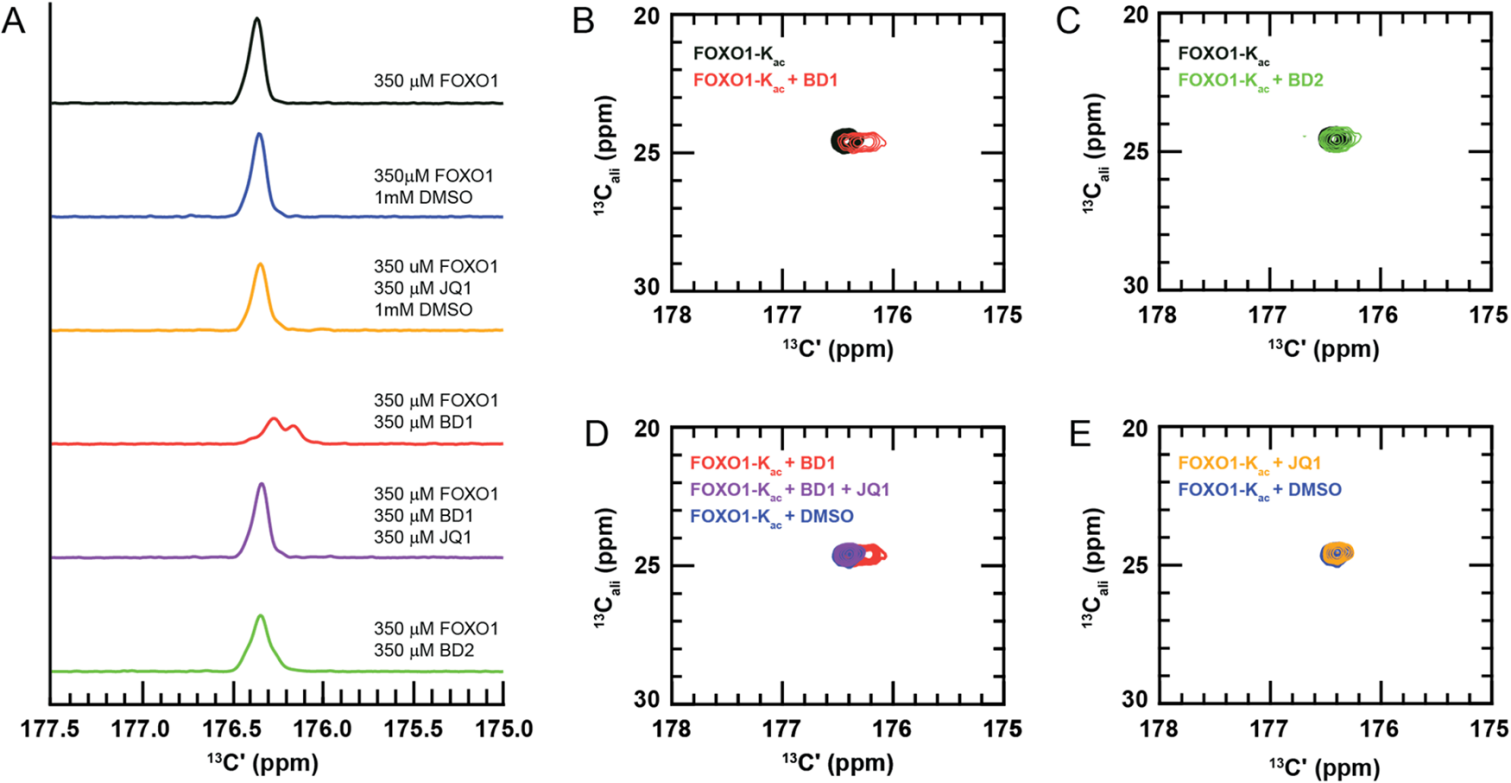
Methyl proton-start [^13^C′,^13^C^ali^]-CaliCO-K_ac_-IPAP reveals chemical shift perturbations of the FOXO1 IDR acetyllsine resonance upon binding to the first bromodomain of BRD4. (A) The 1D ^13^C′ spectrum of 0.35 mM ^13^C-acetyl FOXO1 IDR displays one resonance for the 2–3 acetyl state (black) that is unchanged by addition of DMSO (blue) or JQ1 in DMSO (yellow). Addition of 0.35 mM BD1 (red) results in resolution of the acetyllysine resonance into two distinct resonances, which is eliminated through addition of JQ1 (purple). No spectral change is observed upon addition of BD2 (green). 2D [^13^C′,^13^C^ali^]-CaliCO-K_ac_-IPAP spectra display the same trends through overlay of (B) FOXO1-K_ac_ in buffer and FOXO1-K_ac_ + BD1, (C) FOXO1-K_ac_ in buffer and FOXO1-K_ac_ + BD2, (D) FOXO1-K_ac_ + BD1 and FOXO1-K_ac_ + BD1 + JQ1 (with DMSO control), and (E) control spectra of FOXO1-K_ac_ + JQ1 and FOXO1-K_ac_ + DMSO. Colors in the 2D spectra match the color definitions for the 1D spectra in panel (A).

In contrast, we saw no chemical shift perturbation upon addition of BRD4 BD2 to acetylated FOXO1 IDR. Prior literature demonstrated binding of a short peptide containing K245ac and K248ac to both BRD4 BD1 and BD2, through [^1^H,^15^N]-HSQC.^47^ For rigor, we purchased synthetic peptides matching the FOXO1 and FOXO3 sequences in the prior study and confirmed by [^1^H,^15^N]-HSQC that both bind to our bromodomain constructs as well, although FOXO1 binding to BRD4 BD2 is associated with fewer resonances lost to line broadening and smaller chemical shift perturbations overall, compared with binding of the same peptide to BD1 (Figure S8). It is noteworthy that the high solubility of the FOXO1 240–255 peptide allowed us to reach a 5:1 mol ratio relative to the bromodomains, through a concentration >1.0 mM for the peptides, suggesting that if similar concentrations were accessible in the [^13^C′,^13^C^ali^]-CaliCO-K_ac_-IPAP spectra reported in Figure 5, BD2 binding may become detectable. In all cases investigated here, the limits of peptide solubility preclude quantitative determination of an equilibrium binding constant, as is common for weak binding systems. The findings herein highlight the importance of access to multiple detection modalities, including site-specific modification and direct detection approaches when interrogating protein–protein interactions involving PTMs, especially within IDRs where sequence context may strongly influence binding. Thus, the ability to reliably monitor lysine acetylation and its functional consequences highlights the utility of these methods for probing biologically relevant protein–protein interactions involving modified IDRs.

Despite visualization of direct binding between the two constructs, there are multiple interpretations of the spectrum which cannot be disambiguated with present data. As there is a mixture of acetyl-states in the sample, it is impossible to differentiate between at least two hypotheses: (1) that the two resonances seen upon binding to BRD4-BD1 represent at least two acetylated lysines which become nondegenerate upon binding, for example due to preferential binding of one or the other, or (2) that a resonance representing the twice acetylated state and thrice acetylated state become nondegenerate upon binding, but each resonance still represents more than one acetylated lysine.

As a first step toward addressing the possibility of mixed BRD4 binding modes on FOXO1 IDR, we constructed a series of five single-point mutants to introduce either K233R, K245R, K248R, K262R, or K265R. All five mutants were acetylated from a single batch of p300. Analysis proceeded by [^13^C′,^13^C^ali^]-CaliCO-K_ac_-IPAP spectroscopy and MALDI-TOF mass spectrometry. In each case, NMR spectroscopy confirmed binding of the acetylated peptide to BRD4 BD1, with similar chemical shift perturbations and the appearance of two resonances in the spectrum (Figure S9). As expected, the FOXO1 K233R sample was acetylated less efficiently than wild type, with the +1 and +2 acetyl states dominant, and retention of the unacetylated peak in the mass spectrum (Figure S9A). In each of the remaining four cases, the distribution of acetylation states changed more fundamentally (Figure S9B–E). To varying degrees, each featured retention of the unacetylated peak in the mass spectrum, along with enhancement of the +3 and +4 acetyl peaks. These results suggest that the sequence context in which lysine residues are presented to p300 is an important contributor to the quality of these peptides as substrates for the enzyme. While intriguing, these results underscore the limitations of the current methods to disentangle site-specific effects, while providing clear motivation for future mechanistic inquiries.

## 4 Conclusions

We report herein the backbone ^13^C/^15^N resonance assignments for a portion of the FOXO1 intrinsically disordered region (residues 226–270 in native FOXO1, or 407–451 in PAX3-FOXO1) present within the oncogenic fusion protein PAX3-FOXO1, which is known to carry acetylation marks that enhance its transcriptional activity. PAX3-FOXO1 is acetylated at a novel site, K233, compared with previous reports of acetylated lysines in native FOXO1. Additionally, BRD4, which colocalizes with PAX3-FOXO1 at active superenhancers, has been hypothesized to stabilize acetylated PAX3-FOXO1 through a direct interaction. Interestingly, BRD4 has not yet been demonstrated to directly bind endogenous FOXO1. While this may be accounted for by increased abundance of BRD4 colocalized at superenhancers in ARMS patient derived cell lines, it is also impossible to rule out that the presence of the endogenous forkhead domain obscures acetyllysine binding sites or that K233 acetylation helps to recruit the bromodomain. Here, we show that the interaction between PAX3-FOXO1 and BRD4 is mediated through the acetyllysines in the FOXO1-conributed IDR and occurs specifically through the first bromodomain of BRD4 in a manner which can be interrupted by the highly selective inhibitor JQ1. Together, these results highlight a need for molecular characterization of acetylated transcription factors with BRD4.

## Supplementary Material



## Notes

Nine figures providing disorder predictions, full sweep-width spectra, mass spectrometry data, views of baseline resolution in acetyllysine selective spectra, and secondary NMR chemical shifts (PDF)

## Abbreviations

ARMS: alveolar rhabdomyosarcoma
BD: bromodomain
BME: betamercaptoethanol
BMRB: biological magnetic resonance bank
BRD4: bromodomain-containing protein 4
CSP: chemical shift perturbation
FOXO1: forkhead box O1
IDR: Intrinsically disordered region
IPTG: isopropyl β-d-1-thiogalactopyranoside
LB: Luria broth
MALDI-TOF: matrix-assisted laser desorption/ionization-time-of-flight
NMR: nuclear magnetic resonance
PAX3: paired box 3
P-TEFb: positive transcription elongation factor b
PTM: post-translational modification
re-CHIP seq: rechromatin immunoprecipitation followed by sequencing
