# Supplementary material for: PAX3-FOXO1 Contacts BRD4 through Its Acetylated Intrinsically Disordered Region
Source: Biochemistry. 2026 Jun 12;65(13):2066–76. doi: 10.1021/acs.biochem.6c00040 (PMC13348024; doi:10.1021/acs.biochem.6c00040)
Supplement: Supplementary file 1 [file bi6c00040_si_001.pdf]

Supporting Information

PAX3-FOXO1 contacts BRD4 through its acetylated intrinsically disordered region

*Olivia A. Fraser[1,2,†], Madeline N. Schleicher[1, †], Maya L. Pagano[1] and Scott A. Showalter[1,2] **

[1]Department of Chemistry, The Pennsylvania State University, University Park, PA 16802

[2]Center for Eukaryotic Gene Regulation, Department of Biochemistry and Molecular Biology, The Pennsylvania State University, University Park, PA 16802

†Authors contributed equally to the body of work.

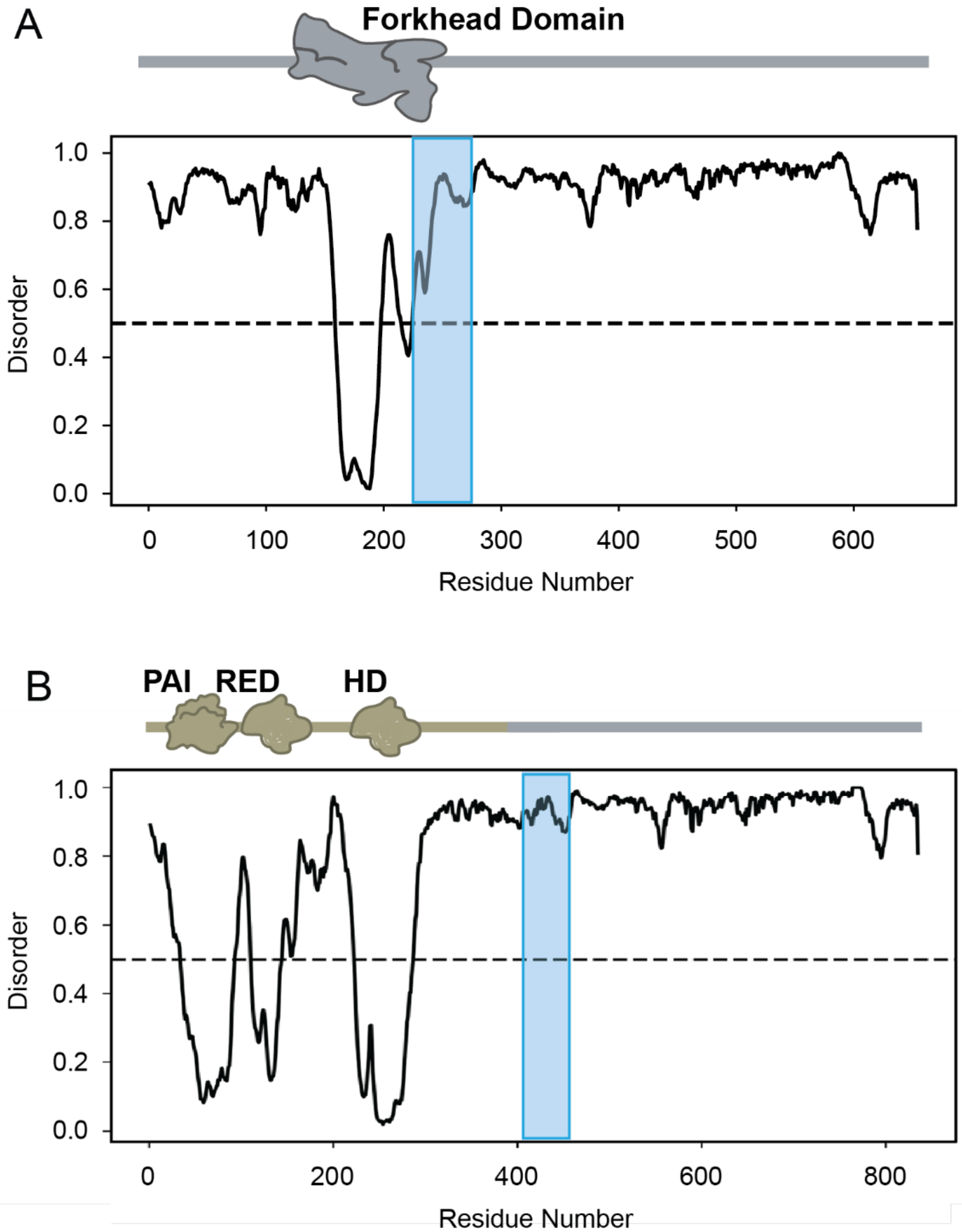

Figure S1. Metapredict traces of FOXO1 and PAX3-FOXO1 predict that the intrinsically disordered nature of the FOXO1-derived region is retained in the fusion protein. (A) Domain schematic and Metapredict profile for human FOXO1. (B) Domain schematic and Metapredict profile of the PAX3-FOXO1 fusion protein. In both panels, a Disorder score > 0.5 (above the dashed line) is indicative of predicted disorder, while scores < 0.5 (below the dashed line) indicate the likelihood of a folded domain. The blue box in both panels indicates the sequence corresponding to the FOXO IDR peptide investigated in this study.

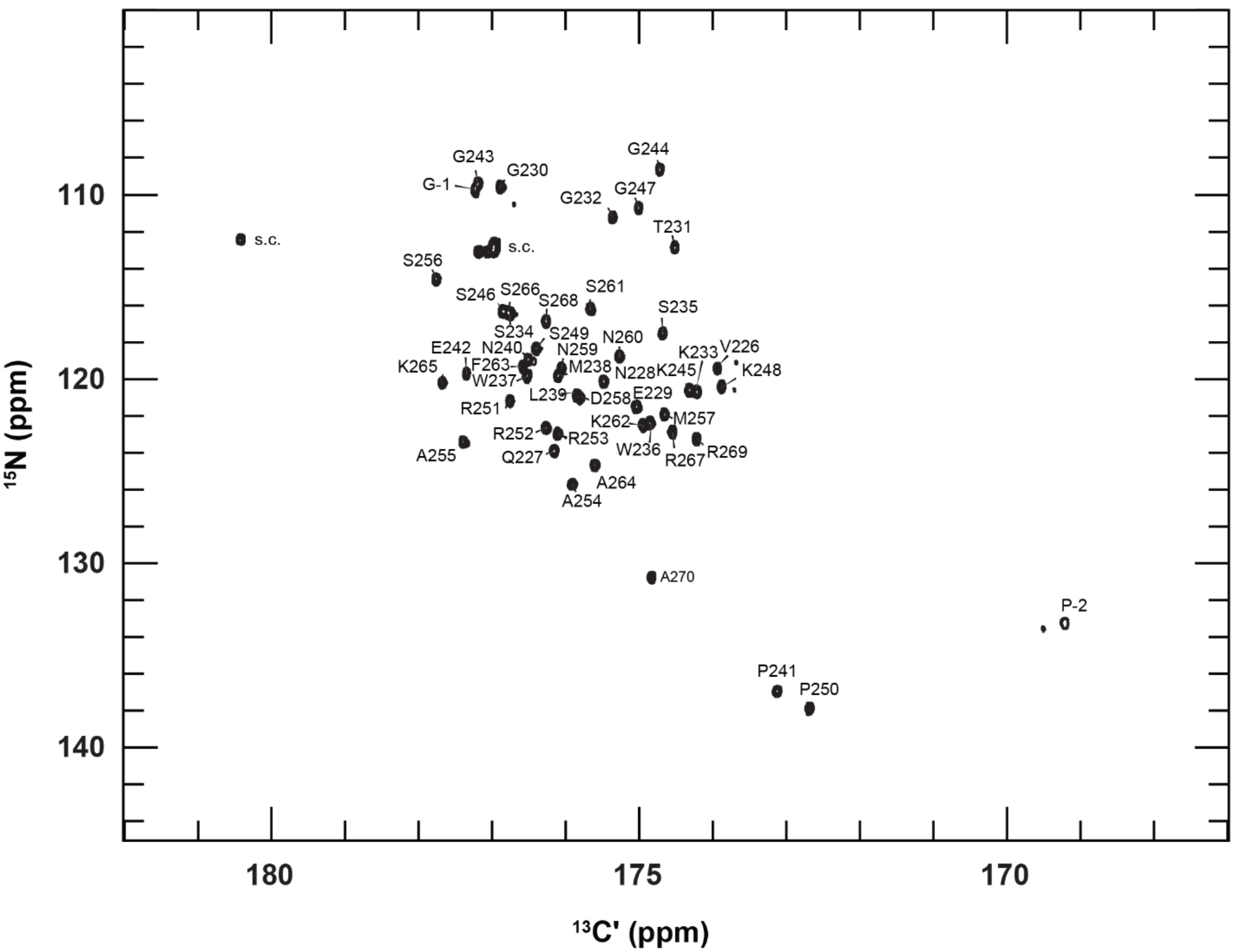

Figure S2. Annotated ^{13}C,^{15}N-CON spectrum of unacetylated FOXO1 IDR. Residue assignments are annotated to reflect the residue contributing the nitrogen atom to the C′N bond. Note that the two assignable resonances from the N-terminal Gly-Pro-Gly cloning artifact are designated as P-2 and G-1, respectively. Resonances corresponding to the side chains of Asn and Gln residues are labeled as "s.c." for clarity.

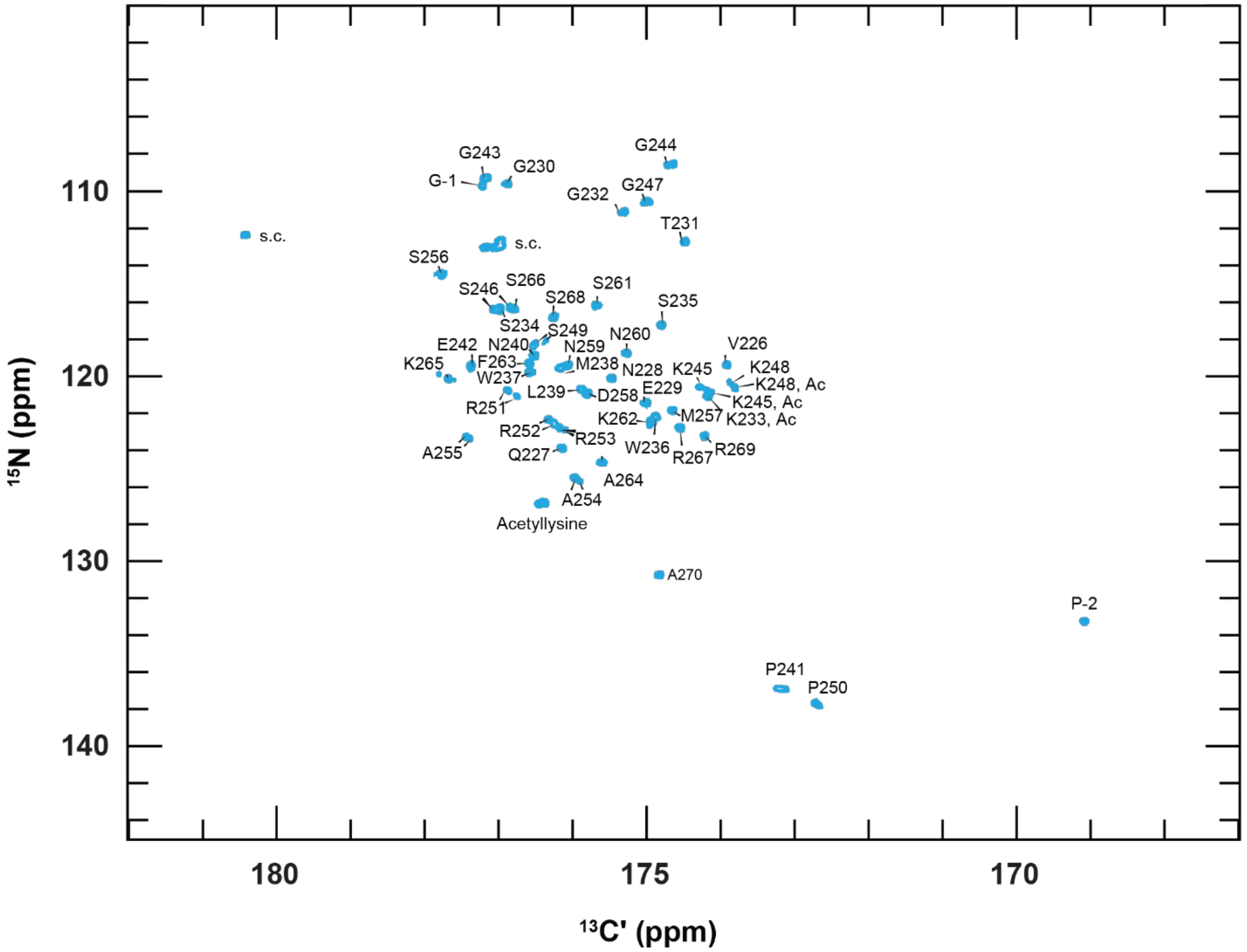

Figure S3. Annotated ^{13}C,^{15}N-CON spectrum of p300-acetylated FOXO1 IDR. Residue assignments are annotated to reflect the residue contributing the nitrogen atom to the C′N bond. Note that the two assignable resonances from the N-terminal Gly-Pro-Gly cloning artifact are designated as P-2 and G-1, respectively. Resonances corresponding to the side chains of Asn and Gln residues are labeled as “s.c.” for clarity.

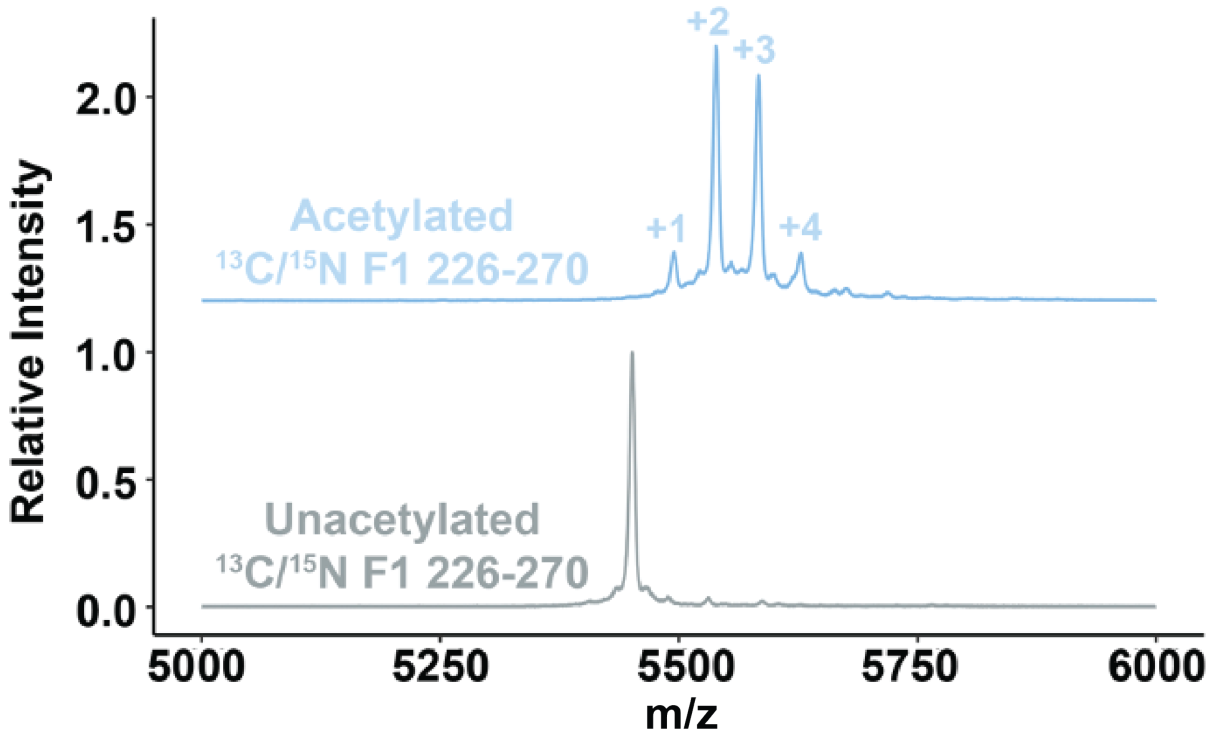

Figure S4. Verification of FOXO1 IDR acetylation by p300 through MALDI-TOF mass spectrometry. The mass spectrum of unacetylated (grey) and acetylated (pale blue) FOXO1 IDR are displayed with an artificial relative intensity offset for clarity. The mass spectrum of acetylated FOXO1 IDR confirms stoichiometric conversion of the peptide to acetylated forms, with transfer of +1, +2, +3, and +4 all represented in the final state. The dominant species after p300 treatment are a mixture of peptides with +2 and +3 acetyllysine residues, respectively.

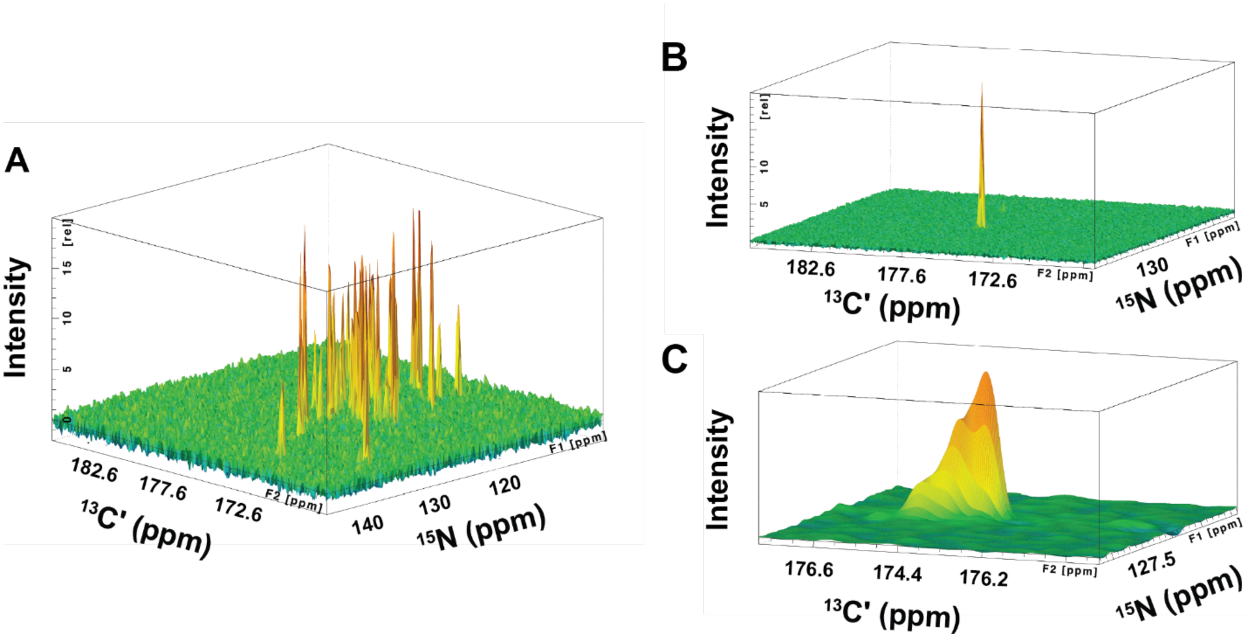

Figure S5. Stacked representations of 2D [^{13}C′, ^{15}N]-CON and [^{13}C′,^{15}N]-CON-K$_{ac}$ spectra demonstrate multiple partially resolved acetyllysine resonances. (A) Stacked plot of the [^{13}C′, ^{15}N]-CON for acetylated FOXO1 IDR provides a visual reference for the signal-to-noise for comparison with the acetyllysine selective experiments. (B) [^{13}C′,^{15}N]-CON-K$_{ac}$ of acetylated FOXO1 IDR displayed to the same sweep width limits as the non-selective CON display in panel A, demonstrating the outstanding backbone signal suppression provided by this experiment. (C) Zooming in on the only significant spectral feature in the [^{13}C′,^{15}N]-CON-K$_{ac}$ shows the existence of up to three partially resolved resonances, consistent with the MALDI-MS demonstration that acetylation of FOXO1 IDR under control of the p300 acetyltransferase domain reaches an endpoint mixture of +2 and +3 acetyllysine residues.

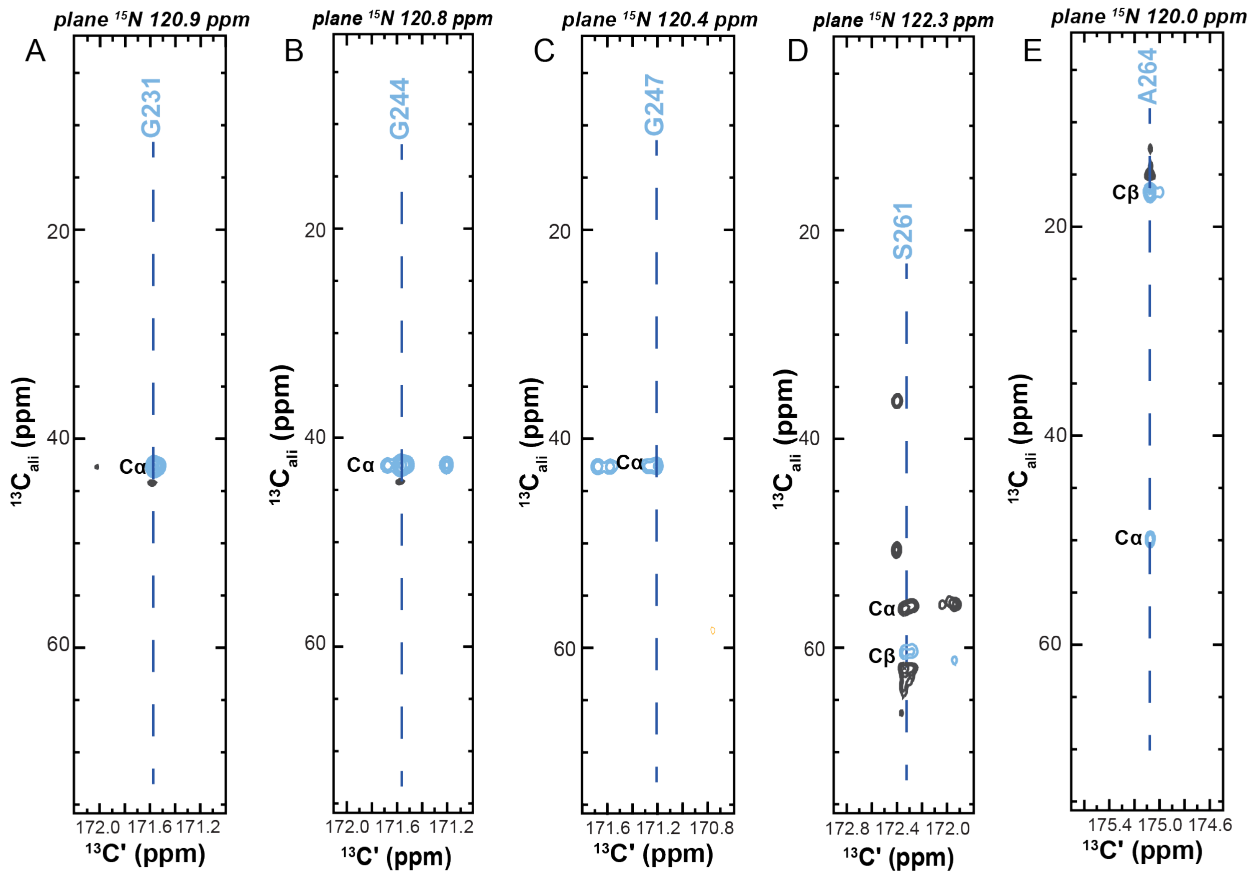

Figure S6. Planes from a three-dimensional (H)CCCON-IPAP spectrum acquired on FOXO1 IDR subjected to acetylation conditions. The aliphatic ^{13}C dimension is populated with resonances corresponding to the aliphatic carbon spin system of the residue preceding each lysine. Thus, for (A) K233, (B) K245, and (C) K248, a single resonance is observed, corresponding to a glycine α-carbon. A more extended "walk along the backbone" differentiates these three Gly-Lys pairs. (D) For K262, two resonances corresponding to the serine α- and β-carbon uniquely assigns this Ser-Lys pair in the sequence. (E) For K265, two resonances corresponding to the alanine α- and β-carbon uniquely assigns this Ala-Lys pair in the sequence. Each panel displays a strip taken at the ^{13}C chemical shift of the carbonyl carbon from a lysine residue, in the ^{15}N plane corresponding to the chemical shift of the backbone amide nitrogen in the residue preceding the lysine. Positive intensity contours are displayed in blue, while negatively phased intensity contours are displayed in black.

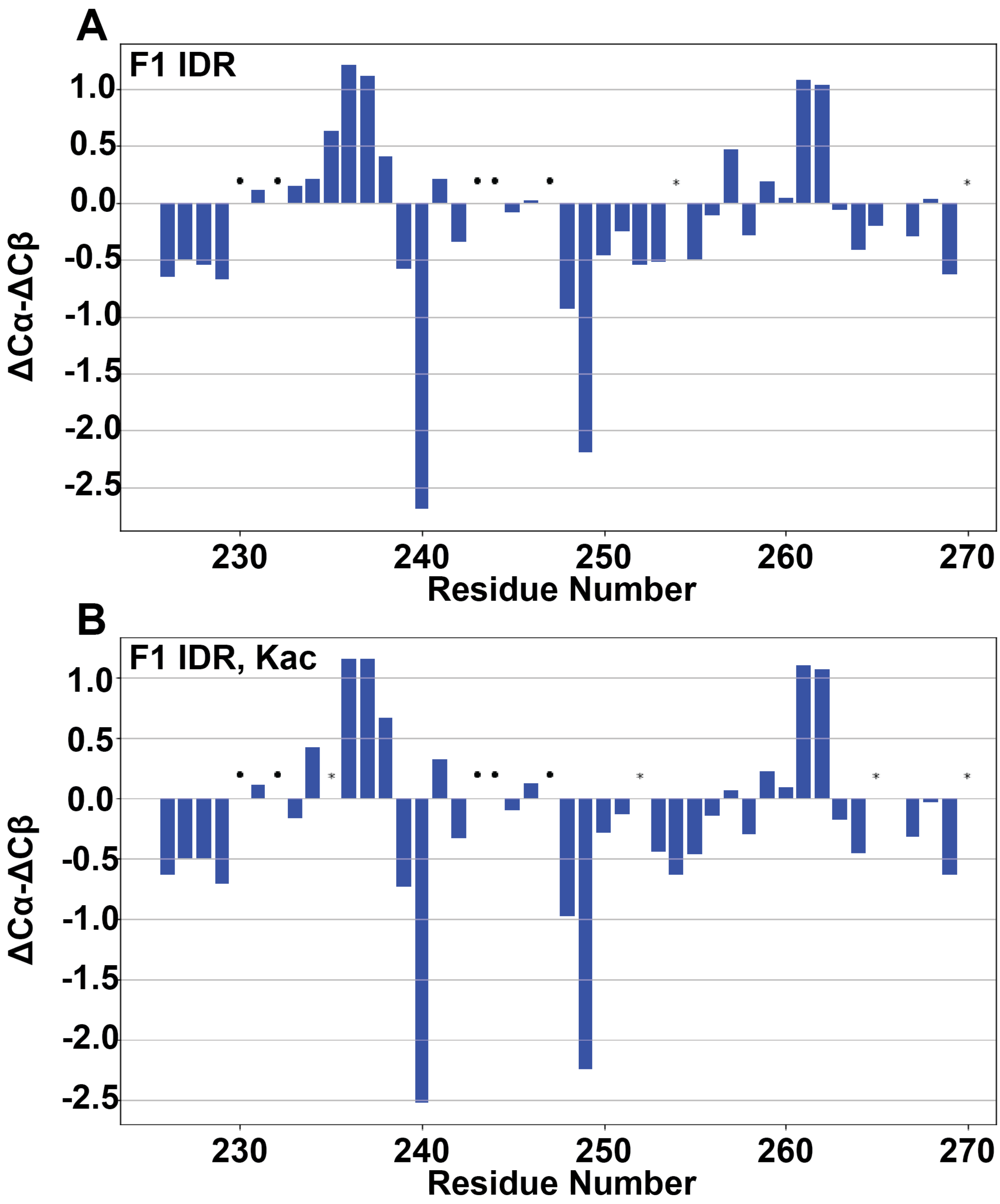

Figure S7. NMR estimation of ensemble secondary structure plotted as the difference between the deviation from random coil for $^{13}C_{\alpha}$ and $^{13}C_{\beta}$. (A) Per-residue chemical shift deviation for wild-type FOXO1 IDR and (B) per-residue chemical shift deviation for FOXO1 IDR K233R. Black circles (·) indicate Gly residues, which have no $^{3}C_{\beta}$. Residues marked with an asterisk (*) had insufficient recorded chemical shifts to calculate the chemical shift difference.

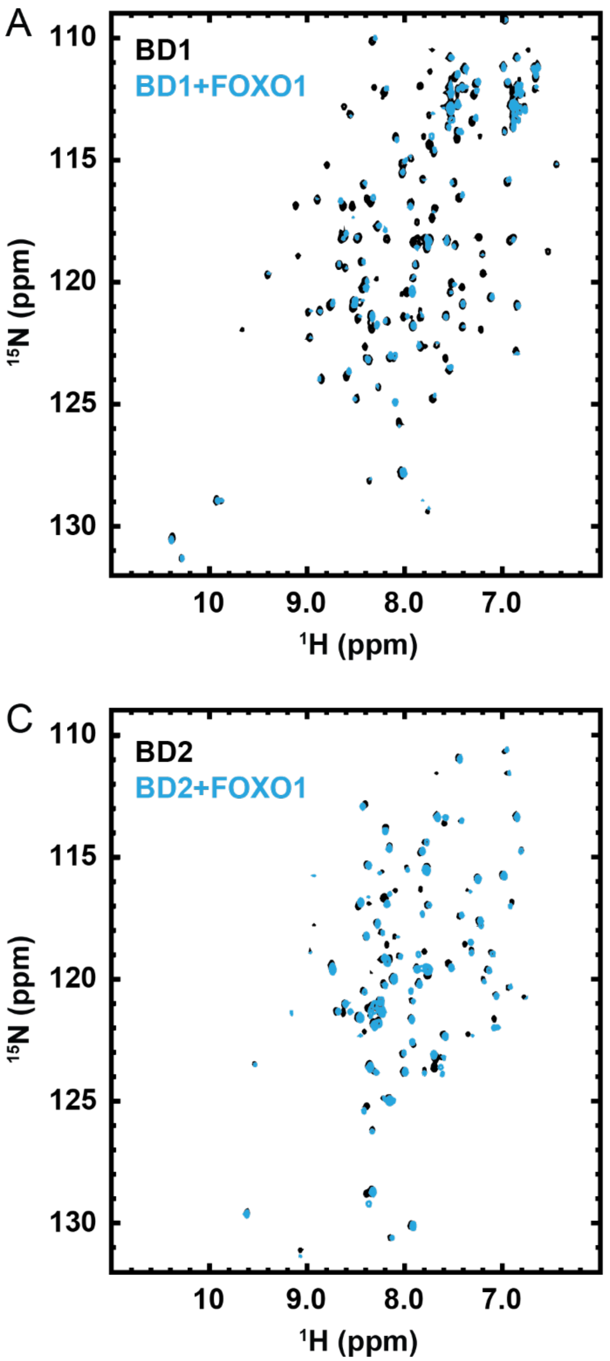

Figure S8. [^{1}H,^{15}N]-HSQC spectra confirm binding between BRD4 bromodomains and acetylated peptides derived from FOXO1 and FOXO3. Spectral overlays in each panel display (A) BRD4 BD1 (black) and BRD4 BD1 bound to FOXO1 240-255, K245ac, K248Ac (blue); (B) BRD4 BD1 (black) and BRD4 BD1 bound to FOXO3 240-255, K242ac, K245Ac (blue); (C) BRD4 BD2 (black) and BRD4 BD2 bound to FOXO1 240-255, K245ac, K248Ac (blue); and (D) BRD4 BD2 (black) and BRD4 BD2 bound to FOXO3 240-255, K242ac, K245Ac (blue). Although the residue numbers in the human sequences of FOXO1 and FOXO3 are identical, as is the placement of the Kac residues in the sequences, the full amino acid sequences are not identical. In all panels, the FOXO1 or FOXO3 peptide was present in five-fold molar excess.

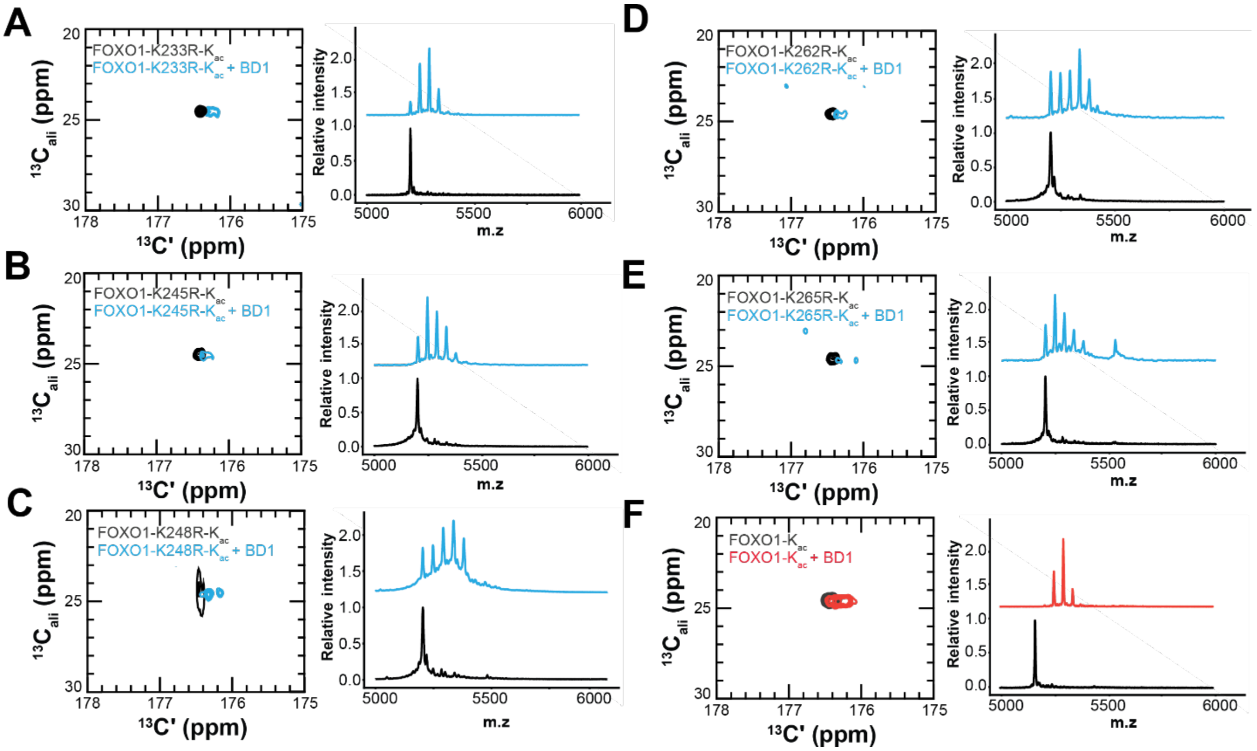

Figure S9. Mutational analysis of FOXO1 IDR acetylation and binding to BRD4 BD1. Constructs employed include (A) FOXO1 K233R, (B) FOXO1 K245R, (C) FOXO1 K248R, (D) FOXO1 K262R, (E) FOXO1 K245R, and (F) wild-type FOXO1 IDR. In all panels, overlays of the unbound (black) and BRD4 BD1 bound (color) [$^{13}C'$,$^{13}C^{ali}$]-CaliCO-K_{ac}-IPAP are displayed on the left, with MALDI-TOF mass spectra of untreated (black) and p300-treated FOXO1 (color) on the right. Data in all six panels were acquired using a single preparation of p300.
